# A computational structural study on the DNA-protecting role of the tardigrade-unique Dsup protein

**DOI:** 10.1038/s41598-020-70431-1

**Published:** 2020-08-07

**Authors:** Marina Mínguez-Toral, Bruno Cuevas-Zuviría, María Garrido-Arandia, Luis F. Pacios

**Affiliations:** 1 Centro de Biotecnología y Genómica de Plantas (CBGP, UPM-INIA), Universidad Politécnica de Madrid (UPM)-Instituto Nacional de Investigación y Tecnología Agraria y Alimentaria (INIA), Campus de Montegancedo-UPM, Pozuelo de Alarcón, 28223 Madrid, Spain; 2 Departamento de Biotecnología-Biología Vegetal, Escuela Técnica Superior de Ingeniería Agronómica, Alimentaria y de Biosistemas (ETSIAAB), Universidad Politécnica de Madrid (UPM), 28040, Madrid, Spain

**Keywords:** Computational models, Protein structure predictions

## Abstract

The remarkable ability of tardigrades to withstand a wide range of physical and chemical extremes has attracted a considerable interest in these small invertebrates, with a particular focus on the protective roles of proteins expressed during such conditions. The discovery that a tardigrade-unique protein named Dsup (damage suppressor) protects DNA from damage produced by radiation and radicals, has raised expectations concerning its potential applications in biotechnology and medicine. We present in this paper what might be dubbed a “computational experiment” on the Dsup-DNA system. By means of molecular modelling, calculations of electrostatic potentials and electric fields, and all-atom molecular dynamics simulations, we obtained a dynamic picture of the Dsup-DNA interaction. Our results suggest that the protein is intrinsically disordered, which enables Dsup to adjust its structure to fit DNA shape. Strong electrostatic attractions and high protein flexibility drive the formation of a molecular aggregate in which Dsup shields DNA. While the precise mechanism of DNA protection conferred by Dsup remains to be elucidated, our study provides some molecular clues of their association that could be of interest for further investigation in this line.

## Introduction

The remarkable ability of tardigrades to survive environmental extremes has attracted the attention of researchers in biology and biotechnology. Tardigrades, also known as water bears, are a specific phylum (Tardygrada) which includes about 1,300 species found in terrestrial, freshwater and marine habitats^1–3^. They are essentially aquatic invertebrates with lengths between 0.1 and 1 mm. Terrestrial tardigrades require to be surrounded by a thin film of water to maintain skeleton functions and oxygen uptake through cuticle. In the absence of water, tardigrades enter a dehydrated state called anhydrobiosis in which they are able to survive in nearly complete desiccation for up to 10 years^4–6^.

However, these tiny animals have fascinated scientists not only due to their long-lasting tolerance to desiccation but also because dehydrated tardigrades are able to withstand a remarkable range of physical and chemical extremes. They can survive at temperatures as low as − 272 ∘C or as high as 150 ∘C for a few minutes, and at − 20 ∘C for decades^7–9^. Tardigrades withstand essentially 0 atm in space^10^ or high pressure up to 1,200 atm (pressure at the bottom of the Marianas Trench)^11, 12^. They survive immersion in organic solvent^7, 13^ and exposure to high dose of irradiation^14^, with radiation levels in some species as high as 4,000^15^ or 5,000 Gy^16^. These unique features have led to conjecture that although possible nearby astrophysical events (large asteroid impact, supernovae and γ-ray bursts) could annihilate Earth-based life, the resilience of tardigrades would render absolute sterilisation unlikely^17^. Without facing such catastrophic scenarios, tardigrades have been the first animals that have endured exposure to outer space for 10 days^10^, which makes them invaluable candidates to study adaptation of life to inhospitable conditions^7, 18^.

Although the singular properties of tardigrades have sparked considerable interest in them, the molecular mechanisms that enable their exceptional resistance are still not well understood. The recent discovery that a tardigrade-unique protein named Dsup (damage suppressor) associates with nuclear DNA to protect human cells from damage by X-rays has expanded the range of studies on tardigrades in biotechnology and medicine^14, 19, 20^. The decoded genomes of two tardigrade species, the terrestrial *Ramazzottius varieornatus*^14^ and the freshwater less-tolerant *Hypsibius dujardini*^21^ (now termed *Hypsibius exemplaris*^22^: we hereafter use this new denomination) have also expanded the scope of these studies. The compared analysis of these two genomes is providing clues on potential gene mechanisms associated with their different response to desiccation and radiation^23–25^, and is allowing researchers to annotate them in search for novel mechanisms associated to the extremotolerance^26^.

After determining the high-quality genome sequence of *R. varieornatus*, one of the most radiotolerant tardigrades, Hashimoto et al. characterised the Dsup gene (UniProt accession number P0DOW4) encoding a 445-residue protein that suppresses DNA damage induced by X-ray in human cultured cells^14, 19^. Cells expressing Dsup also showed a significant reduction in DNA fragmentation upon exposure to hydrogen peroxide, which generates hydroxyl radicals, one of the reactive oxygen species (ROS) produced by indirect radiation effects. Although the precise mechanism of DNA protection by Dsup was not elucidated, sequence-based analyses led these authors to propose that the protein associates with nuclear DNA in a non-specific manner, physically shielding DNA from direct radiation and ROS damage^19^. More recently, Chavez et al. have reported evidence that Dsup binds to nucleosomes, protecting chromosomal DNA from hydroxyl radicals-mediated DNA cleavage in vitro^25^. These authors found a sequence region in Dsup with similarity to a core consensus sequence of the nucleosome-binding domain of high mobility group N (HMGN)^25^ proteins. These proteins found only in vertebrates bind to high affinity sites on nucleosomes independently of DNA sequence^27^.

**Figure 1:**
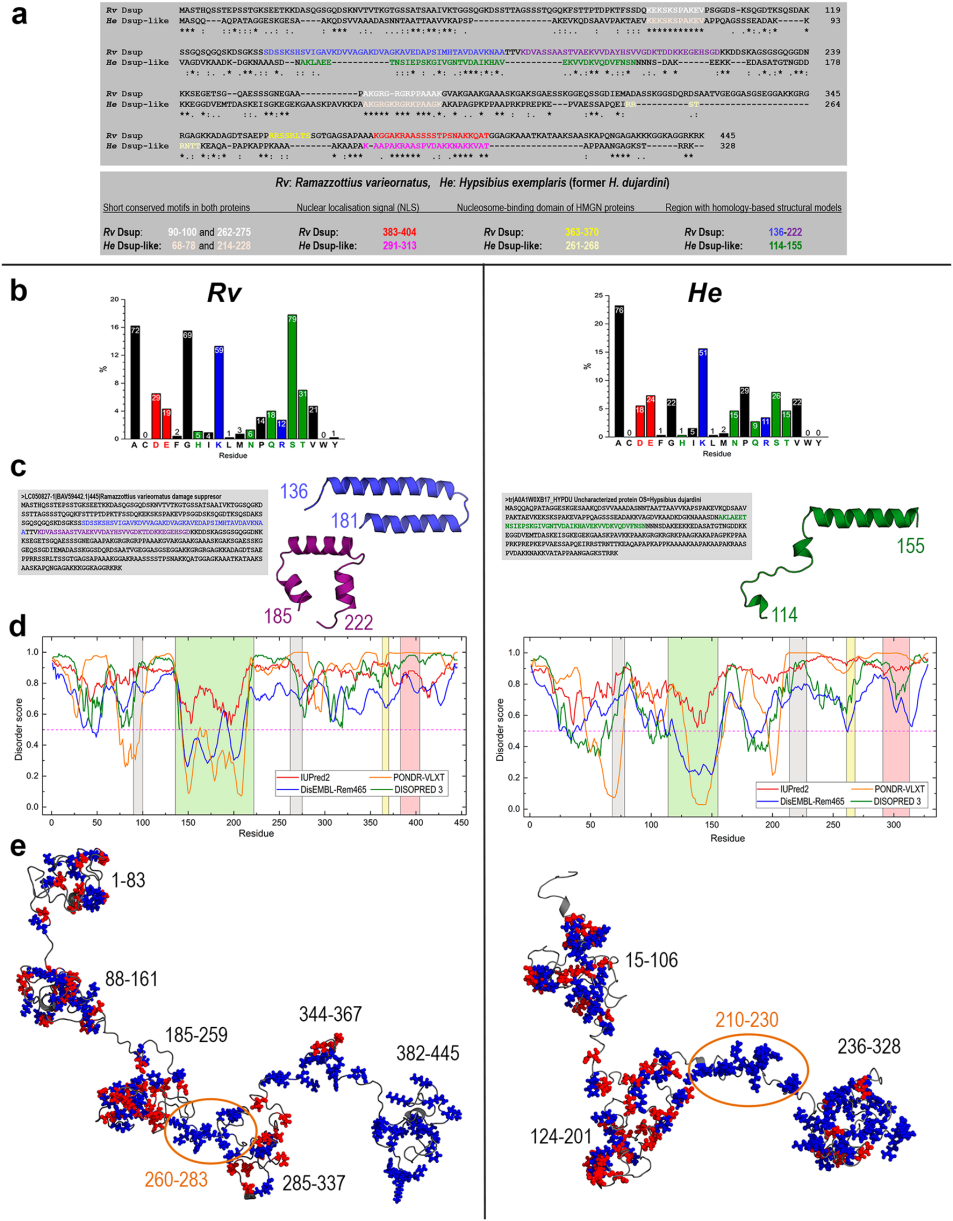
Sequence and structural features of Dsup and Dsup-like proteins. (**a**) Sequence alignment obtained with Clustal-Omega 1.2.4 of the 445-residue Dsup protein from *R. varieornatus* (Rv) and the 328-residue Dsup-like protein from *H. exemplaris* (He) (“*” indicates perfect alignment whereas “:” and “.” indicate sites belonging to groups showing strong and weak similarity, respectively), highlighting the sequence motifs indicated in the box below. (**b**) Bar chart giving the % and number of each amino acid type according to the colour code: black = non-polar, green = polar, blue = positively charged, red = negatively charged. (**c**) Sequence segments with their corresponding structural models obtained by homology-modelling with SwissModel. (**d**) Structural disorder predicted from sequence with four predictors. The disorder score is defined in the range 0–1 with values > 0.5 meaning disorder (DISOPRED 3 gives no values with score < 0.5). Conserved motifs in both proteins, segments modelled by homology-modelling, HMGN-like and NLS sequences are the regions shaded light grey, light green, light yellow and light red, respectively. (**e**) Structural 3D models predicted from sequence by I-TASSER. Charged amino acids are shown as balls (positive in blue and negative in red) with labels indicating the sequence segments in which they accumulate. Regions marked with orange ovals enclose the second conserved motif 262–275 and 214–228 in Rv and He proteins, respectively.

The search for possible Dsup homologues only identified a weakly similar protein from *H. exemplaris*, with 24.5% sequence identity with Dsup^19^. The *H. exemplaris* gene (UniProt accession number A0A1W0XB17) encodes a 328-residue protein annotated without functional information as “hypothetical protein BV898_01301” in the NCBI database. However, nuclear localisation was predicted for both proteins and a putative nuclear localisation signal (NLS) sequence was identified in their C-terminal domains. The alignment also revealed two short sequence motifs strictly conserved between these two proteins, which led to speculate that those motifs could be important for Dsup function^19^. Furthermore, Chavez et al. also found in the protein from *H. exemplaris* a sequence segment closely similar to the conserved core sequence critical for nucleosome-binding of HMGN proteins^25^. On the basis of this known sequence information which is summarised below and as it is also suggested by the novel structural features which are presented here (see Fig. 1), it thus seems reasonable to consider the protein from *H. exemplaris* a “Dsup-like” protein. This label will be used hereinafter to distinguish it from the “Dsup” protein from *R. varierornatus*.

In this work, we focus on Dsup presenting a “computational experiment” aimed to explore molecular details of the direct DNA-Dsup interaction. To this end, we used molecular modelling, analysis of Poisson-Boltzmann electrostatic potentials (PB-EPs) and their electric field, and all-atom molecular dynamics (MD) simulations. We applied these computational approaches to study the following molecular systems: (i) the isolated Dsup protein, (ii) the complex of one Dsup molecule with DNA, and (iii) the complex of two Dsup molecules with DNA. With this last complex, we intended to explore a first case of multiple binding of Dsup to DNA as proposed by experimental results^25^. Nonetheless, the size of the resulting system (more than 750,000 atoms) made its study with all-atom MD simulations being at the limit of what is possible even with the availability of a supercomputer such as that used in this work. As shown below, nearly the entire Dsup molecule is an intrinsically disordered protein that features a high proportion of charged amino acids with a preferential accumulation of positive charges in the C-terminus. In this regard, our findings demonstrate that electrostatic effects play a crucial role in the Dsup-DNA interaction and that the electric field between both molecules has a great strength. Our results also show that the gradual proximity to DNA modulates changes in the disordered motion of several regions of Dsup (mainly at the C-terminal segment) towards a shielding-like association. This report must be taken as a first piece of information regarding structural details on the assumed protective role of Dsup in shielding DNA. To the best of our knowledge, the computational study here presented is the first work aimed to elucidate atomistic details of the possible dynamic modes of action of tardigrade proteins.

## Results

### Dsup is an intrinsically disordered protein as evidenced by disorder prediction and structural modelling

The alignment between Dsup and Dsup-like sequences displayed in Fig. 1a shows the location of the conserved segments reported before: two short (11 and 15 amino acids) identical motifs, the 22-amino acid NLS sequence located in the C-terminal region, and the 8-amino acid motif of the HMGN nucleosome-binding domain. The amino acid composition of both sequences reveals features of structural interest (Fig. 1b). In Dsup, the sum of small non-polar (69G + 72A) and polar (79S + 31T) amino acids amounts to 56.4% of total. There is a large number of charged residues with dominance of positive (59K + 12R) over negative (29D + 19E) amino acids, which together account for 26.7% of total and yield a net charge + 23. In Dsup-like, there is also a majority of small non-polar (22G + 76A) residues but there are far less small polar (26S + 15T) amino acids so that all small residues now amount to 42.4% of total. The sum of charged positive (51K + 11R) and negative (18D + 24E) residues accounts for 31.7% of total with a net charge + 20. Common features of both sequences are that they have (i) no cysteines, (ii) almost no aromatic residues, and (iii) very few non-polar amino acids with long side chains (4I + 1L in Dsup and 5I + 1L in Dsup-like). Since cysteines are usually involved in disulfide bond formation and non-polar, especially aromatic amino acids favour hydrophobic aggregation (bulky tryptophan is absent in both proteins), the very low number of these order-promoting residues together with the high number of small and charged amino acids in these two proteins is consistent with their intrinsically disordered nature shown below.

The alignment in Fig. 1a also shows the equivalent location of a segment consisting of 83 amino acids in Dsup and 42 amino acids in Dsup-like for which we obtained local homology-based structures (Fig. 1c). Even though we did not expect to obtain a homology-based structural model of the entire proteins, we did try this approach to find out whether homology modelling was possible for at least some regions. Swiss-Model delivered for Dsup two small α-helical models for the sequence segments 136–181 and 185–222 (Fig. 1c left), using as templates a region of murine catenin-α-2 protein (PDB id 4ONS) and a region of human annexin A8 protein (PDB id 1W45), respectively. For Dsup-like, Swiss-Model predicted for the segment 114–155 a model composed of an α-helix and two short 3_10_ helices (Fig. 1c right), using as template a region of a bacterial dehydrogenase (PDB id 1XDW). Interestingly, Hashimoto et al. reported an in silico analysis of the Dsup sequence in which an α-helical region was predicted for the 142–206 segment whereas no secondary structure was predicted for the remaining sequence (except a few tiny β-strands)^14, 19^. Although these authors did not address disorder prediction, they speculated that Dsup “…might function with a flexible structure rather than in a rigid form, e.g., as a physical shield of DNA” (page 6 in Ref.^19^).

We used several disorder predictors and their results (Fig. 1d) are quite conclusive: except for a sequence region ~ 140–220, three of the four methods predicted for Dsup a disordered structure and the remaining method, IUPred2, predicted in that region a lower disorder score (region shaded light green in Fig. 1d left). Most disorder predictors are knowledge-based methods. PONDR is a neural network trained on known disordered sequences and its associated VLXT algorithm incorporates information from X ray-characterised disordered regions. DisEMBL and DISOPRED3 are trained on structural data from known disordered regions such as non-assigned electron densities defined as Remark 465 in structure files or loop/coils with high thermal factors. In addition to structural information, DISOPRED3 also uses data on sequences known to be disordered though they have no structure. In contrast, IUPred2 predicts disorder by computing interaction energies between all amino acid pairs in the sequence. Its basic assumption is that disordered proteins have an amino acid composition that precludes favourable inter-residue interactions to stabilise a well-defined structure. IUPred2 uses a fast and reliable statistical potential devised to compute amino acid pairs interaction energies without relying on databases, being thus methodologically different to any other disorder predictor. Despite the distinct approaches underlying these disorder predictors and the secondary structure predictor used in Ref.^19^ for Dsup, they all remarkably agree with our modelling results. These findings can be summarised stating that Dsup is an intrinsically disordered protein (IDP) and that it is possible that the dynamic conformational changes that characterise IDPs would show transient α-helical local segments in the 140–220 region. Similar results were found for Dsup-like: disorder is predicted for most of the sequence except the segment ~ 115–155 (shaded light green in Fig. 1d right) for which three of the four methods again predict no disorder and IUPred2 predicts a lower disorder score. In addition, two small regions ~ 50–70 and ~ 175–210 are now predicted to have no disorder by two methods. As for the sequence segments strictly conserved in Dsup and Dsup-like (Fig. 1a; regions shaded light grey in Fig. 1d), all methods agree in predicting a slightly higher disorder for the second motif (262–275) than for the first one (90–100), and they both flank the central region (shaded light green in Fig. 1d).

In agreement with disorder predictions, the 3D model structures obtained with I-TASSER for the complete sequences (Fig. 1e) predict in fact that both proteins are IDPs. Given that IDPs exist as a dynamic ensemble of conformations that largely vary with time without a single equilibrium structure, these I-TASSER models must be viewed as just one of the possible conformations. As shown below for Dsup, it is the dynamic evolution of the structure what provides the essential information on its behaviour with regard to the interaction with DNA. The disordered structure of Dsup shows charged amino acids clustered in separate regions (Fig. 1e left). While five of them have also acidic residues, two regions have exclusively basic residues: the 260–283 segment (4K + 3R) and the C-terminus (14K + 3R), which has a positive charge + 17. As discussed below, it is this latter positive segment the one that moves preferentially towards DNA, which agrees with the conjecture by Hashimoto et al. that the C-terminus of Dsup and Dsup-like proteins would be involved in DNA association^14, 19^ The Dsup-like model also shows charged residues clustered in regions (Fig. 1e right). There is now a single region with only basic residues: the 210–230 segment (7K + 3R). In contrast, the C-terminal region (15K + 8R) has also six acidic residues, which yields a local positive charge + 17, the same as that of Dsup.

Positive and negative charges are found to be scattered over the entire structures of both Dsup and Dsup-like except in neatly separated regions with accumulation of positive residues (discounting these regions, the net charge is just − 1 in both proteins). This finding could be rationalised by assuming that the constant motion of a disordered chain permits charge balance as an overall stabilising factor, while the existence of separated regions with overwhelming dominance of positive charges suggests that an external factor such as union to DNA is needed to stabilise them. It is worth noting that apart from the C-termini, the segment with exclusively basic residues (marked with orange ovals in Fig. 1e) contains the second of the two conserved motifs between the proteins. While no function has been proposed yet for those motifs, this finding provided by the structural models might suggest a putative role that is presented below after discussing the results of the MD simulations.

It is known that the expression of genes encoding tardigrade-specific IDPs is upregulated in response to drying^28^. A large number of these IDPs have been identified in *H. exemplaris* and the ability of 11 cytosolic abundant heat soluble proteins to vitrify has been demonstrated in vitro and when expressed in yeast^28^. It has been suggested that if other desiccation-tolerant organisms rely on trehalose to adapt to drying, tardigrades would rely on these IDPs to survive nearly complete dessication^28^. These findings agree with the reported properties of late embryogenesis abundant (LEA) proteins, other desiccation protectants that use their disordered flexible structures to protect other molecules by shielding them or to form a material that supports glass transitions during desiccation^29^. Therefore, the suggestion that a disordered protein might function as a flexible structure shielding DNA from irradiation damage seems reasonable and is indeed demonstrated below. The unexpectedly low sequence similarity between Dsup from *R. varieornatus* and Dsup-like from *H. exemplaris* would thus be a hint that they have been under weak selective pressure during evolution. This is a known feature of IDPs as amino acids in disordered regions may change without the physical constraint to maintain a definite structure^30–32^. To summarise this section, Dsup and Dsup-like share: (i) a similar IDP profile, (ii) a central region with potential secondary structure in the form of helical motifs, flanked by two short sequence segments strictly conserved, (iii) a predicted 3D model that shows a similar disordered structure with clusters of charged amino acids, (iv) a separate C-terminal cluster with accumulation of positively charged residues that includes the conserved NLS sequence, (v) a similar cluster of positive amino acids that includes the second of the two strictly conserved sequence motifs, (vi) a similar net positive charge, (vii) an identical net charge − 1 obtained upon excluding the clusters of positive charges. Together with the previously identified conserved sequence segments, our findings strongly suggest that Dsup and Dsup-like may have a common function in which disorder is paramount.

### The initial geometries of Dsup-DNA complexes show a significant electrostatic complementarity

**Figure 2:**
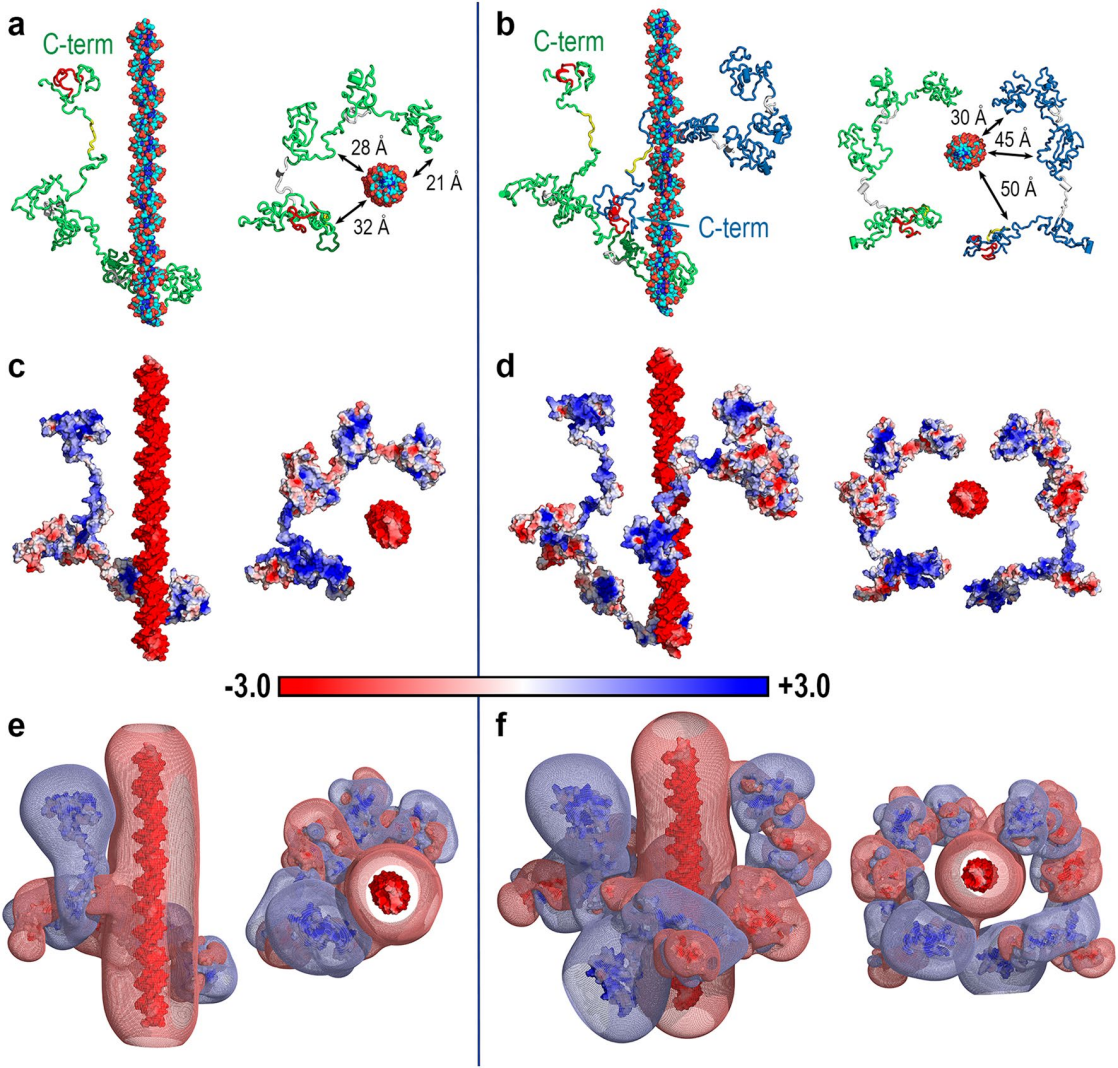
Structure and Poisson-Boltzmann electrostatic potential of the initial geometries of Dsup-DNA complexes. (**a**) Side (left) and top (right) views of the initial geometry of the Dsup-DNA complex. The protein is shown as a green ribbon with regions corresponding to conserved sequence segments coloured as in Fig. 1a for Rv Dsup. DNA is shown as ball-and-sticks with P atoms in orange, O atoms in red, N atoms in blue and C atoms in cyan. Minimum distances between DNA and the three segments of Dsup closer to DNA are indicated in the top view. (**b**) Same views for the (Dsup)_2_-DNA complex. The first Dsup molecule hereby named DsupA is shown as in (**a**) and the second molecule hereby named DsupB is shown as a blue ribbon with the same colouring of sequence segments used in Fig. 1a for Rv Dsup. Minimum distances between DNA and the three segments of the second Dsup molecule closer to DNA are indicated in the top view. (**c**) Side and top views of the PB-EP mapped onto the surface of protein and DNA in the Dsup-DNA complex. (**d**) Same views as those shown in (**c**) for the (Dsup)_2_-DNA complex. The bar gives the colour code for the range of PB-EP values (in *kT*/*e* units) in these mappings. (**e**) PB-EP + 0.1 (light blue mesh) and -0.1 (light red mesh) isosurfaces superimposed on the surfaces shown in (**c**) for the Dsup-DNA complex. (**f**) Same views for the (Dsup)_2_-DNA complex.

Aimed to study the dynamic behaviour of Dsup, we performed MD simulations on the isolated protein and on two protein-DNA complexes with stoichiometry 1:1 and 2:1. The size of the solvation box required to address these systems with all-atom MD calculations gives rise to a very large number of atoms: 414,174 in the 1:1 complex and 755,424 in the 2:1 complex, from which only 9,561 and 15,503 atoms, respectively, correspond to protein and DNA. In the 1:1 case, we set up the initial geometry of the complex (Fig. 2a) by placing one Dsup molecule so that it could interact with almost an entire DNA ideal model (see “Methods”). Since experimental evidence indicates that Dsup interacts with free DNA^14, 19^ and with nucleosomes^25^ independently of the nucleotide sequence, we modelled the DNA in the most frequent B conformation based on a randomly generated 57-nucleotide sequence. Given that the dynamics probed by MD calculations modifies the geometry of both protein and DNA due to interactions between them and with water, this ideal structure of DNA is just a starting point for the dynamic study. The initial separation between Dsup and DNA was wide enough to allow the MD simulation to bring both molecules closer together but not far enough to need an excessive number of simulation steps just to place the two molecules at interaction distance (Fig. 2a). In the 2:1 case, we added to this 1:1 geometry a second Dsup molecule at a slightly greater distance from DNA and less parallel orientation with respect to DNA (Fig. 2b) to enable the MD simulation to move the second Dsup molecule under the effects from both DNA and the first Dsup molecule (see distances in Fig. 2).

Mapping of PB-EPs onto the surfaces of Dsup and DNA reveals an overall electrostatic complementarity in both 1:1 (Fig. 2c) and 2:1 complexes (Fig. 2d). The markedly negative EP on the surface of DNA, overwhelmingly dominated by the negative phosphate groups in the 57 nucleotides of each chain, contrasts with the dominantly positive EP on the surface of Dsup. The spatial domains defined by the PB-EP -0.1 and + 0.1 isosurfaces in these initial geometries (Fig. 2e,f) show that the negative isosurface uniformly located around DNA complements large patches of the positive isosurfaces that arise from the proteins. As shown below, the dynamic evolution of these systems brings Dsup and DNA closer together by reinforcing this electrostatic complementarity.

**Figure 3:**
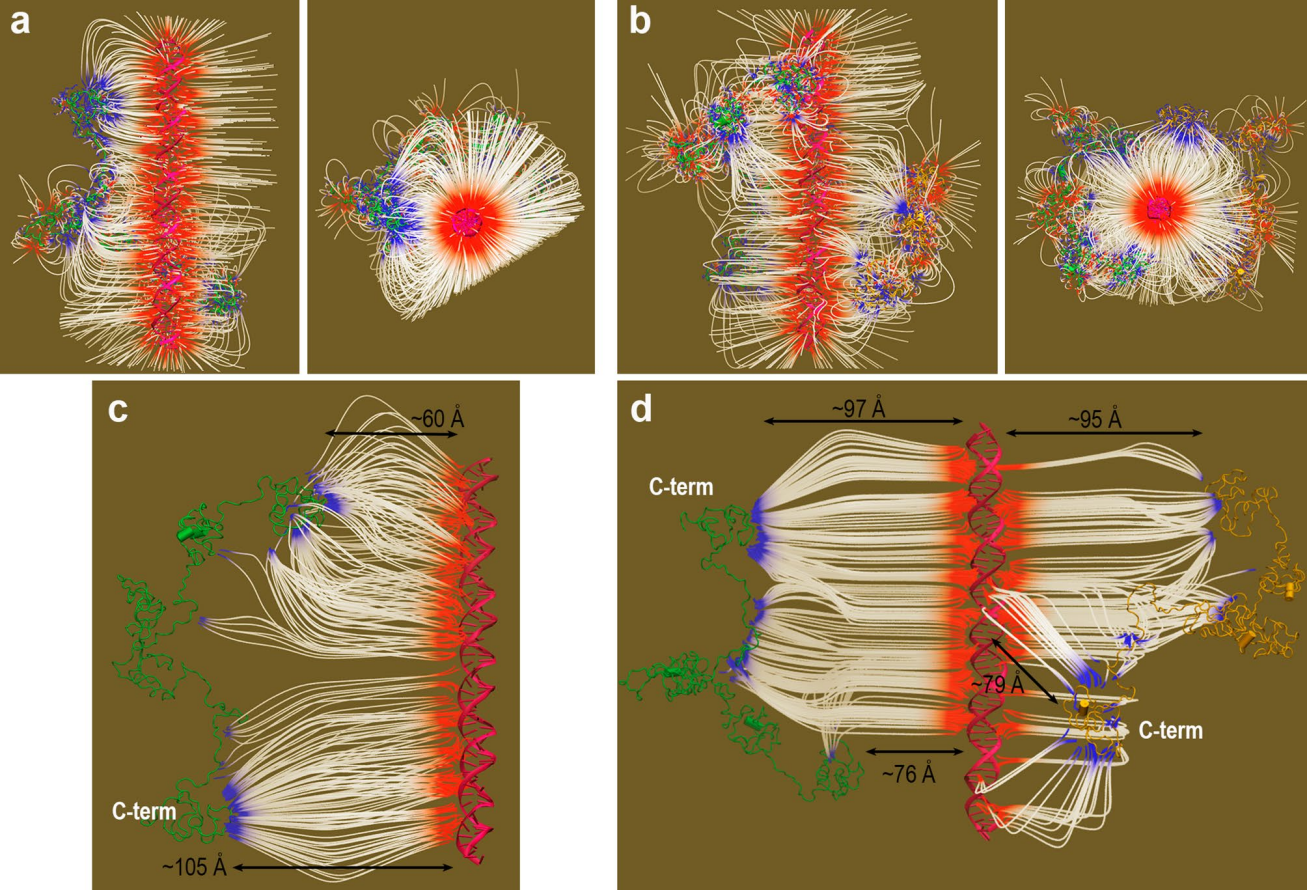
Electric field *E* in the initial geometries of Dsup-DNA complexes. (**a**) Side (left) and top (right) views of field lines for ***E*** = 6 *kT*-equivalent units in the initial geometry of the Dsup-DNA complex shown in Fig. 2a. (**b**) Same views as those used in (**a**) for the (Dsup)_2_-DNA complex. (**c**) Side view of field lines for ***E*** = 3 *kT*-equivalent units in a geometry of the Dsup-DNA complex resulting from moving Dsup further apart from DNA in the geometry shown in (**a**). (**d**) Side view of field lines for ***E*** = 3 *kT*-equivalent units in a geometry of the (Dsup)_2_-DNA complex resulting from moving both Dsup molecules further apart from DNA in the geometry shown in (**b**). Field lines in images (**a**)–(**d**) start on (+) charges and end on (−) charges depicted by blue and red colours, respectively.

The electric field generated by the molecular EP *V*(**r**) is the negative gradient of the potential, ***E*** = −∇*V*(**r**). Field lines of ***E*** are shown in Fig. 3a,b for the initial geometries of the two Dsup-DNA complexes displayed in Fig. 2. Additional field lines are shown in Fig. 3c,d for two geometries obtained upon moving the proteins further apart from DNA by a horizontal translation in the orientations shown for the two complexes in Fig. 3a,b. Electric field lines start on positive charges and end on negative charges, which are represented in Fig. 3 by blue and red colours, respectively. *V*(**r**) is given in *kT*/*e* units (*k* being the Boltzmann’s constant, *T* the absolute temperature and *e*, the electron charge) so that at room temperature, 1 *kT*/*e* = 0.0258 J/C = 0.0258 V. Since the electric field has units of Volts/length, one *kT*-equivalent unit of electric field ***E*** in molecular systems is equal to 0.0258 V/Å = 2.58 × 10^8^ V/m. The strength (i.e., the magnitude) of the electric field at any spatial region is proportional to the density of field lines in that region. Therefore, the electric field shown in Fig. 3 for initial geometries in which Dsup and DNA are separated a considerable distance indicates that the great number of charges in both molecules generates intense electric fields. Even at the large intermolecular distances in Fig. 3a,b, these fields are ~ 0.15 V/Å = 1.5 × 10^9^ V/m and ~ 0.08 V/Å = 8 × 10^8^ V/m (Fig. 3c,d). For the sake of comparison, it has been reported that external electric fields of strength ~ 10^9^ V/m accelerate photosynthetic reactions by an order of magnitude and that internal fields also about 10^9^ V/m are generated at active sites of enzymes^33^.

### All-atom MD simulations reveal the distinct local behaviour of regions of Dsup and the dynamic role of the C-terminal domain in the presence of DNA

In this and next sections, we present results from all-atom MD 100 ns simulations for the following systems: isolated Dsup (two trajectories), Dsup-DNA 1:1 complex with (i) the complete Dsup chain, (ii) a chain lacking the C-terminal domain, and (iii) another chain consisting of only this domain, and (Dsup)_2_-DNA 2:1 complex. In the 1:1 complex, the initial geometries of (ii) and (iii) were those of the corresponding segments in the initial geometry of full Dsup. In the 2:1 complex, the two Dsup molecules are hereafter referred to as DsupA and DsupB (see Fig. 2). Since as already mentioned, Dsup is predicted to be intrinsically disordered except perhaps a middle region covering approximately residues 140–220, large dynamic changes are expected along its backbone except perhaps at that middle region. However, it is highly unlikely that large elements of secondary structure could appear in our MD simulations because the local folding processes that would be involved occur at much longer times than those amenable to MD study with very large proteins such as Dsup. Nonetheless, during the constant structural changes of a full IDP it is expected that (a) transient tiny secondary structure elements will appear at small sparse segments, and (b) different backbone regions will exhibit a different mobility.

**Figure 4:**
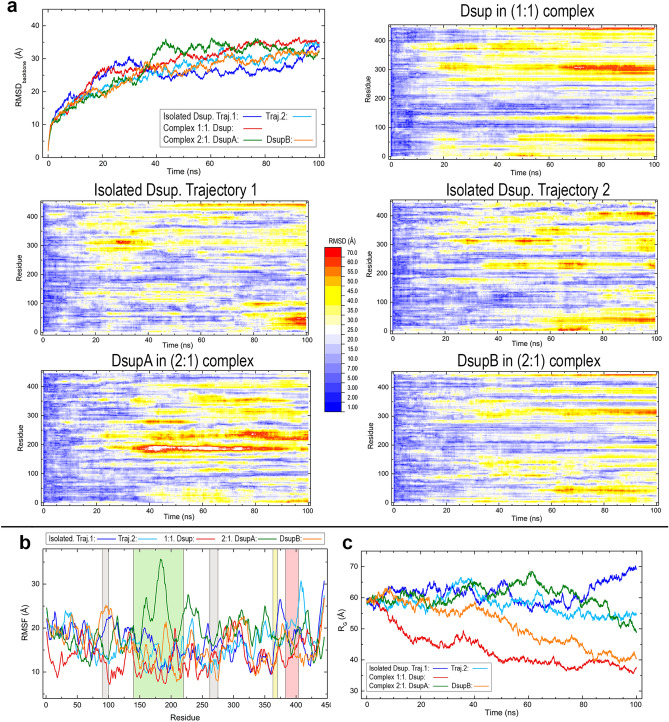
Structural data computed for Dsup along all-atom MD 100 ns trajectories in the systems studied. (**a**) Plot of RMSD values for Dsup protein (first panel) and heatmap plots of RMSD for the 445 residues computed with backbone atoms. (**b**) RMSF plot. Sequence regions shaded in different hues correspond to the sequence segments indicated in Fig. 1d with the same colours. (**c**) Radius of gyration.

The results of root mean square deviations (RMSD) computed with backbone atoms over all-atom 100 ns MD simulations are shown in Fig. 4a, first as a plot for the complete backbone and then as a set of heatmap plots displaying the values of the 445 residues. The disordered motion of the protein as a whole is reflected into the high RMSD of the full chain (first panel in Fig. 4a) which shows a closely similar pattern in all the systems. The mobility measured by the RMSD increases rapidly during the first 10 ns, then increases more smoothly during the next 20 ns and at ~ 40 ns is stabilised at a large value RMSD ~ 30 Å in all the trajectories. This result suggests a consistent dynamic behaviour for the disordered structure of our model of Dsup protein. Heatmap plots giving the RMSD of all residues along the simulations show a distinct pattern for different segments in the sequence. A common feature in all the trajectories is the large mobility of some sequence regions such as the first N-terminal 50 amino acids, a region around residue 300 and most part of the C-terminal residues beyond position ~ 350, which includes both the nucleosome-binding sequence of HMGN proteins (363–370) and the NLS sequence (383–404). The first segment 90–100 strictly conserved in Dsup and Dsup-like shows a low mobility in most trajectories while the second strictly conserved segment 262–275 shows a higher mobility in the DNA complexes than in the two isolated trajectories. It is worth noting that these features are in reasonable agreement with (a) the disorder prediction in Fig. 1d, where it is seen that the two conserved segments do have different disorder scores in most methods, and (b) the 3D models in Fig. 1e where the second conserved segment is found to be part of a positively charged cluster which could be involved in DNA interactions. As for the central region for which local homology-based models were obtained (136–222), all the trajectories except that of DsupA in the 2:1 complex show smaller RMSD values in this sequence region ~ 150–250 than in most of the remaining residues. These plots suggest that the two Dsup molecules in the 2:1 complex influence each other as they display a qualitatively different behaviour in that middle region.

The latter result is all the more apparent in the spatial fluctuations of residues measured by the root mean square fluctuation (RMSF) displayed in Fig. 4b. In fact, DsupA and DsupB molecules in the 2:1 complex exhibit a markedly different behaviour particularly in the region ~ 150–250 in which DsupA has far greater fluctuations than DsupB. Dsup in the 1:1 complex shows an overall lesser fluctuation than that found in the remaining systems, a result which can be explained by the closeness between large structural segments of the protein and DNA that appear early in the dynamics predicted by MD calculations as discussed below.

Even though the radius of gyration (R_G_) might not be considered a representative property in an IDP, its trends in the Dsup systems are worth to note. At the final simulation time, R_G_ shows a decreasing tendency in the three Dsup proteins in complex with DNA whereas it is stabilised or even increases in the two trajectories of isolated Dsup (Fig. 4c). In addition, the values of R_G_ in two of the three cases in which Dsup interacts with DNA are about half of the values in the two isolated Dsup cases. This result suggests a propensity towards a more compact structure in the presence of DNA.

**Figure 5:**
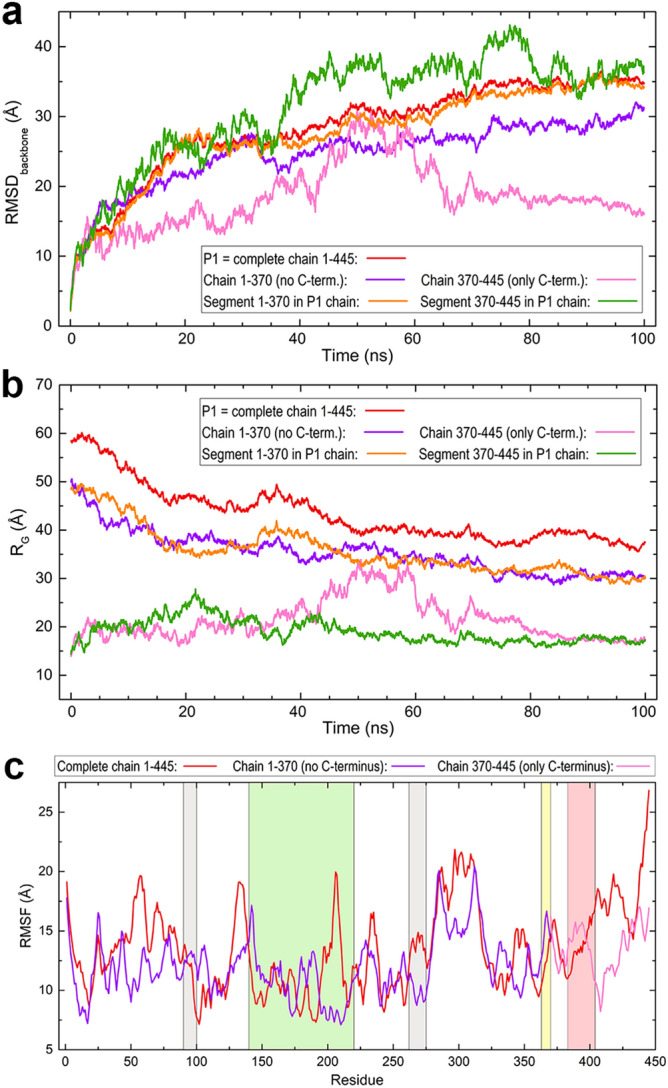
Structural data computed along all-atom MD 100 ns simulations for different chains of Dsup in the Dsup-DNA 1:1 complex. (**a**) Plot comparing RMSD computed with backbone atoms for (i) the complete chain 1–445, (ii) for a chain 1–370 lacking the C-terminal domain, (iii) for a chain 370–445 consisting only of this C-terminal domain, and (iv, v) for the two corresponding segments in the complete Dsup chain. (**b**) Plot comparing values of the radius of gyration for chains (i)–(v). (**c**) RMSF for chains (i)–(iii). Sequence regions shaded in different hues correspond to the sequence segments indicated in Fig. 1d with the same colours.

In previous experimental reports, the C-terminal domain of Dsup has shown to be required and sufficient for DNA binding^14, 19^. Aimed to explore the dynamic role of this domain in the interaction with DNA, we performed additional MD calculations in the 1:1 complex for a Dsup chain 1–370 lacking the C-terminal domain and for another chain 370–445 consisting of this domain alone. Comparisons of RMSD, R_G_ and RMSF for these chains and for the corresponding segments in the full chain, all in complex with DNA, are depicted in Fig. 5. The main results are the following. (i) RMSD values of chain 1–370 are very similar to those of the complete chain but become smaller from ~ 40 ns until the end of the simulation while showing a similar profile (Fig. 5a). As expected from their relative lengths, R_G_ values of chain 1–370 are smaller than those of the complete protein, with a similar profile along the whole simulation (Fig. 5b). (ii) The RMSD and R_G_ values of chain 370–445 (C-terminal domain alone) are obviously much smaller than those of the two other chains, but there are now dramatic changes. From initial time until ~ 40 ns and then from 60 ns until the end of the simulation, both RMSD and R_G_ show a relatively flat profile. However, between ~ 40 and 60 ns the isolated C-terminal domain has RMSD even greater than those of chain 1–370 (Fig. 5a) and R_G_ values are similar (Fig. 5b), results that may at first sight appear surprising if one considers the different chain lengths involved in the comparison. (iii) RMSD of the C-terminal domain in the full chain also shows a marked increase at ~ 40 ns, remains at large values until ~ 80 ns and then decreases becoming similar to that of the complete protein (Fig. 5a). R_G_ of this segment in the full chain shows a featureless flat profile revealing that its size remains practically unchanged (Fig. 5b). (iv) The segment 1–370 in the full protein follows a noticeably similar trend in both RMSD and R_G_ values to that of the complete protein whereas the C-terminal domain in the protein accounts for markedly larger RMSD fluctuations from ~ 40 ns until ~ 80 ns (Fig. 5a), and a different R_G_ tendency especially before ~ 40 ns (Fig. 5b). (v) The backbone of chain 1–370 displays overall lower RMSF values than the complete chain and lacks the large fluctuations that full Dsup shows in several segments (~ 50–90, ~ 125–135, ~ 195–215 and ~ 295–310). The backbone of the isolated C-terminal domain shows a markedly lower fluctuation than that of the full protein in this domain, which is particularly noticeable in the segment ~ 405–430 just following the NLS sequence (Fig. 5c).

To understand the results on the dynamic behaviour of the isolated C-terminal domain it must be taken into account that its initial geometry was taken from the corresponding 370–445 segment in the model structure of full Dsup, which is a compact positively charged cluster (382–445 in Fig. 1e). This choice was made in order to provide further clues on the interaction dynamics of the C-terminal domain with DNA, as Fig. 5 suggested that this domain in particular has a greater mobility than the rest of the chain for most of the simulation time. The MD trajectory of the chain 370–445 with the C-terminal domain alone reveals the balance between its dynamic motion and charge interaction with DNA. This trajectory is illustrated by the snapshots in Supporting Fig. 1: in what follows, times refer to those snapshots. The domain begins moving as a compact chain (low R_G_) with low mobility (low RMSD) because its initial geometry is a compact cluster (0–8 ns). When it is still far from DNA, the repulsion among its positive charges starts to extend its backbone (increasing R_G_) which in turn provokes a great mobility (increasing RMSD) in water (16–32 ns). While these effects keep on stretching out the chain (largest RMSD and R_G_), one of its ends approaches DNA (40–64 ns). When the attraction between the positive charges of the C-terminal chain and the negative charges of DNA is strong enough because of their closer proximity, the backbone chain compacts (low R_G_) decreasing thus its mobility (low RMSD) until it tightly attaches to DNA (72–100 ns). This dynamics in which the isolated C-terminal domain is packed during a substantial portion of the simulation time gives rise to a lower fluctuation (RMSF) of this segment 370–445 alone than that of the corresponding segment in the full protein, in which it is part of an IDP that strongly influences its mobility (Fig. 5c).

### The dynamic changes of Dsup-DNA distances provide insight into the interaction

**Figure 6:**
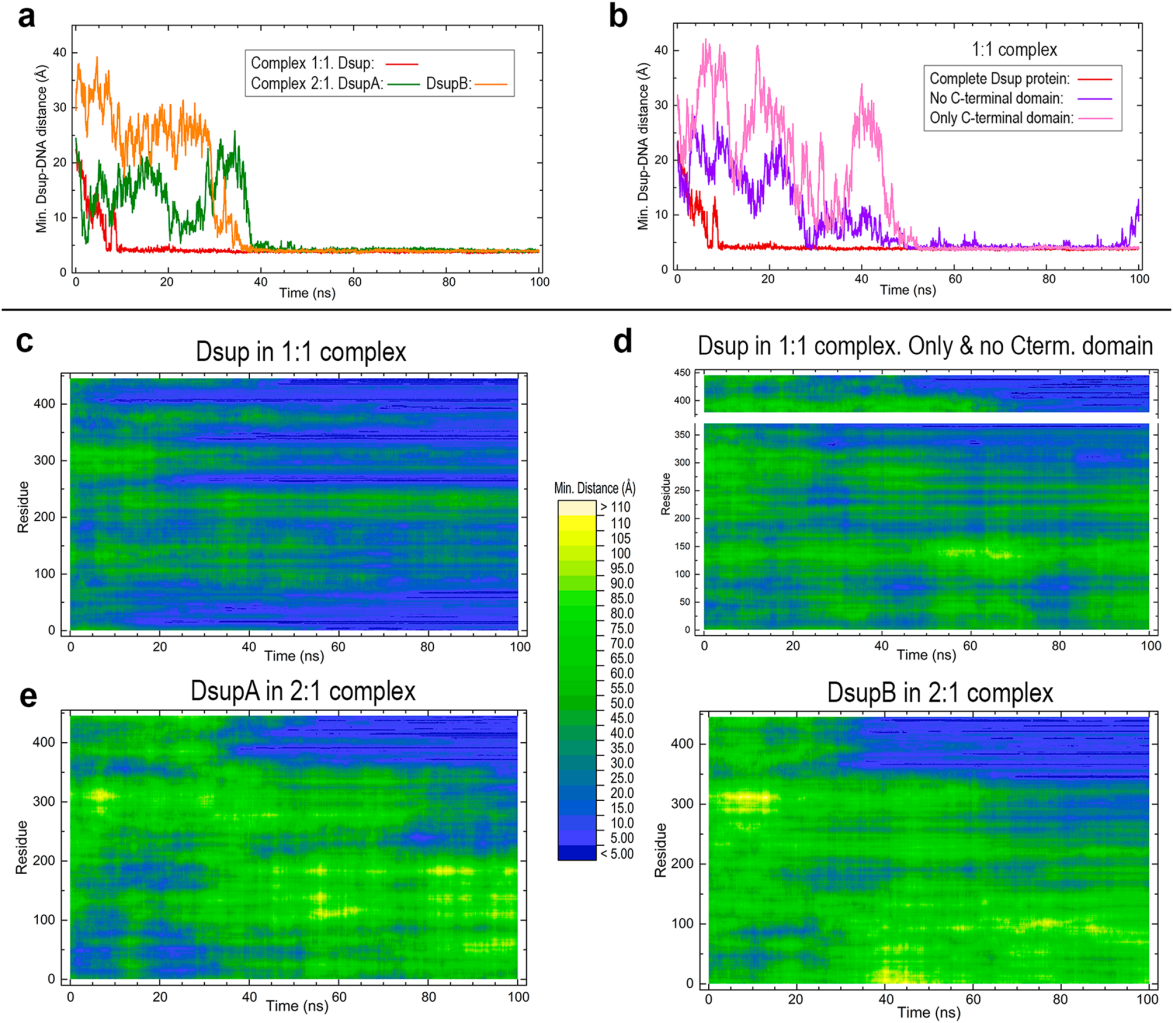
Minimum distances between Dsup and DNA computed along all-atom MD 100 ns simulations for the Dsup-DNA complexes. These are minimum distances between any Cα atom of Dsup and any P atom of DNA. (**a**) Minimum distances computed with complete Dsup chains in the two complexes. (**b**) Minimum distances in the 1:1 complex computed with the complete Dsup chain, with a chain lacking the C-terminal 370–445 domain and with a chain consisting only of this domain. (**c**) Heatmap plot of minimum distances of residues for the complete Dsup chain in the 1:1 complex. (**d**) Heatmap plot for a chain 1–370 without the C-terminal domain (lower part) and for a chain 370–445 composed of only this domain (upper part). (**e**) Heatmap plots for DsupA and DsupB molecules in the 2:1 complex.

**Figure 7:**
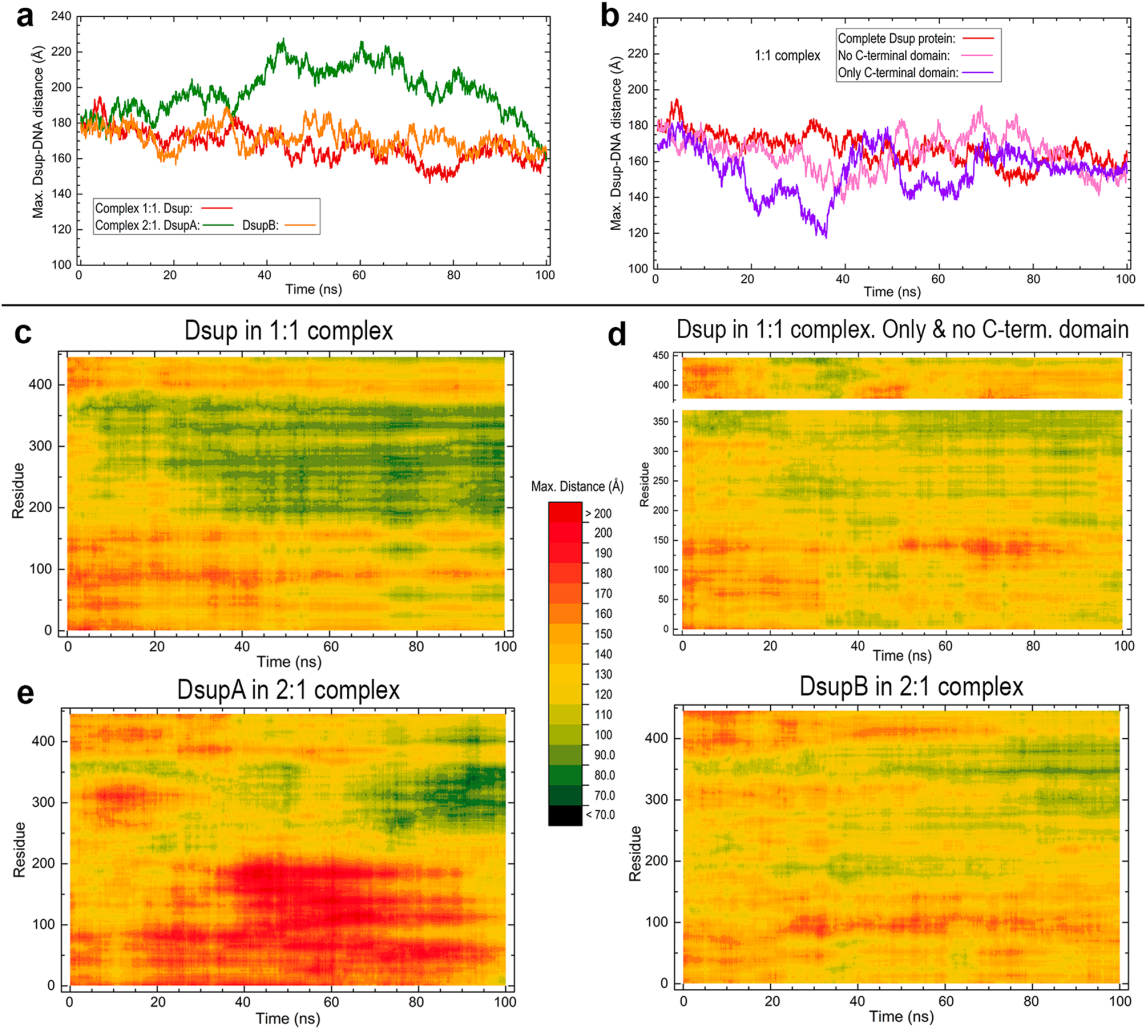
Maximum distances between Dsup and DNA computed along all-atom MD 100 ns simulations for the Dsup-DNA complexes. These are maximum distances between any Cα atom of Dsup and any P atom of DNA. (**a**) Maximum distances computed with complete Dsup chains in the two complexes. (**b**) Maximum distances in the 1:1 complex computed with the complete Dsup chain, with a chain lacking the C-terminal 370–445 domain and with a chain consisting only of this domain. (**c**) Heatmap plot of maximum distances of residues for the complete Dsup chain in the 1:1 complex. (**d**) Heatmap plot for a chain 1–370 without the C-terminal domain (lower part) and for a chain 370–445 composed of only this domain (upper part). (**e**) Heatmap plots for DsupA and DsupB molecules in the 2:1 complex.

The interaction between Dsup and DNA is now analysed by means of the minimum (Fig. 6) and maximum (Fig. 7) distances between both molecules computed over the MD simulations. These distances were calculated at each trajectory frame as the minimum and maximum values among all the distances between Cα atoms in all the protein residues and P atoms in all the nucleotides in the two DNA chains. The minimum distance for the protein as a whole (Fig. 6a) starts to decrease at the very beginning of the simulations and then stabilises at a small value at 9 ns in the 1:1 complex and at 40 ns in the 2:1 complex. Since the initial geometry of the 2:1 complex was prepared by adding a second Dsup molecule (DsupB) to the initial geometry of the 1:1 complex, the initial values of this minimum distance are the same for the 1:1 complex and for DsupA in the 2:1 complex. However, although the two Dsup molecules in the 2:1 complex reach a steady minimum distance to DNA at practically the same time, they take much longer than Dsup in the 1:1 complex. This result is in agreement with the abovementioned mutual influence of both Dsup molecules in the 2:1 complex. The closest distance between Dsup and DNA is in all cases approximately 4 Å (Fig. 6a), revealing a tight and stable interaction. The comparison of minimum distances of the full protein with those of the chain lacking the C-terminal domain and those of this domain alone provides further insights into the role of the C-terminal region on the interaction with DNA (Fig. 6b). In the absence of the C-terminal domain, the 370-residues chain reaches a minimum value for the first time at 30 ns but almost immediately increases again and it is not until 50 ns that it recovers minimum values although with small increases which are even larger at the end of the simulation (Fig. 6b). The great mobility of the isolated C-terminal domain discussed above reflects into the large oscillations of its minimum distance to DNA until decreasing at 40 ns, followed by a large increase between 40 and 60 ns, to finally reach a steady minimum distance ~ 4 Å (Fig. 6b).

Heatmap plots in Fig. 6 give the minimum distances from all Dsup residues to DNA along the MD simulations. It is noticed that the approximately hundred C-terminal residues invariably achieve closest proximity to DNA from simulation times ~ 40 ns in all cases. However, the rest of Dsup chain displays a different behaviour. In the 1:1 complex, lower values of minimum distances to DNA also appear in the second half of the simulation in other regions of the complete chain (Fig. 6c) such as the approximately hundred residues-long N-terminal segment and large regions between positions ~ 250 and ~ 350. All these regions reach their closest approach to DNA at about 40 ns as well. After this time, no minimum distances longer than 50 Å are found in Fig. 6c, displaying a tight intermolecular interaction between Dsup and DNA (see Supporting video 1). The role of the C-terminal domain on the proximity to DNA is dramatically demonstrated by comparing this Fig. 6c with Fig. 6d, both referring to the same 1:1 complex. The isolated C-terminal domain (upper part of plot d) shows a pattern very similar to that of the two Dsup molecules in the 2:1 complex (Fig. 6e) but not to that of complete Dsup (Fig. 6c): middle values > 50 Å of minimum distances during the first 40 ns and then low values < 15 Å during the remaining 60 ns (Fig. 6d). Again, the chain lacking the C-terminal domain (lower part of plot d) shows an overall pattern rather different from that observed in the corresponding 1–370 segment of the complete protein (Fig. 6c) and more similar to that of both Dsup molecules in the 2:1 complex (Fig. 6e). The distribution of the minimum distances in this 2:1 complex shows that the closest approach to DNA now appears exclusively in the C-terminal region in the two Dsup molecules (Fig. 6e). If one looks at their relative positioning with respect to DNA in the initial geometry of the MD simulation (see Fig. 2b), these heatmaps suggest that each Dsup molecule moves towards DNA as a whole during the first 40 ns while still away from DNA and then orient their C-termini closer to DNA thus remaining during the next 60 ns (see Supporting video 2). This result is consistent with the repeated observations on the changes occurring at these times discussed above for Fig. 6a. Finally, the segment 262–275 (the second of the two strictly conserved motifs in Dsup and Dsup-like), is close to DNA in all heatmap plots in Fig. 6, which agrees with being inside a cluster of positively charged amino acids (260–283 in Fig. 1e, left).

Maximum Dsup-DNA distances give a complementary picture (Fig. 7). Values computed with the complete proteins (Fig. 7a) remain with little changes during the first 20 ns in both complexes. But whereas these distances smoothly decrease after this time in a similar fashion for Dsup in the 1:1 complex and for DsupB in the 2:1 complex, they increase and remain at large values for DsupA in the latter complex. In all cases, these distances converge to a common value at the end of the simulation. The comparison of maximum distances of complete Dsup with those of chain 1–370 lacking the C-terminal domain and those of this domain alone suggests that the impact of this C-terminal region on the maximum separation from DNA is lesser than on the minimum distances to DNA (Fig. 7b). This is expected as maximum distances would reveal regions that specifically separate or indirectly remain apart from DNA and the MD results discussed above demonstrate that the C-terminal domain is the Dsup region which attaches to DNA in all cases. Chain without the C-terminal domain exhibits some fluctuations in the maximum distances during the first 40 ns, which means that in the absence of this domain the mobility of the disordered chain increases along this time. However, these values are smaller than those of the complete chains and from 40 ns on, all distances converge to a same maximum value about 160 Å (Fig. 7a,b).

Heatmap plots in Fig. 7 showing the maximum distances from DNA of all Dsup residues reveal that the larger values along the most part of the simulation correspond in all cases to several segments in the N-terminal 150 residues. In the 1:1 complex, the smaller values appear at a large region between positions 150 and 350 (Fig. 7c). As anticipated by Fig. 7b, the role played by the C-terminal domain on the separation from DNA (Fig. 7d) is weaker than that played on the proximity to DNA (Fig. 6d). The main effect is now that the lack of this domain (lower part of Fig. 7d) gives place to slightly longer maximum distances than those in the complete chain in the region 150–350 (Fig. 7c). In contrast, the values for the isolated C-terminal domain (upper part of Fig. 7d) are rather similar to those observed for the full chain (Fig. 7c). Interestingly, greater differences are found for the two Dsup molecules in the 2:1 complex (Fig. 7e). Whereas DsupB shows no particularly large maximum values along time, DsupA displays during most of the simulation a large segment spanning the first half of the sequence that shows values significantly greater than the other half. This result suggests that in the presence of DsupB, the N-terminal half of the disordered chain of DsupA moves away from DsupB as far as possible while tightening the interaction with DNA of its other half, revealing again that two Dsup molecules interacting with DNA can influence each other regarding their dynamic behaviour.

To summarise this section, the dynamic evolution of the different parts of Dsup in its interaction with DNA presents a distinct behaviour in the two complexes analysed. In the 1:1 complex, the greatest part of Dsup shows small values of both minimum and maximum distances to DNA, suggesting that most of the protein is closely interacting with DNA. The role of the C-terminal domain studied in this complex seems paramount in allowing the complete IDP to get closer to DNA. In the 2:1 complex, the presence of an additional Dsup molecule introduces an effect on the other Dsup molecule which was above anticipated on the basis of the RMSD heatmaps (Fig. 4a). In fact, the qualitatively different behaviour in N-terminal and middle regions noticed in the distances heatmaps (Figs. 6e, 7e) reveals that DsupA and DsupB influence each other. It must be mentioned that on the basis of in silico analyses of the sequence, Hashimoto et al. speculated that the N-terminal and middle regions of Dsup could play a role to relieve possible adverse effects induced by formation of large Dsup-DNA complexes^14, 19^. This conjecture is in remarkable agreement with our findings derived from the analysis of the dynamic changes in protein-DNA distances occurring during the MD simulations. Finally, as far as changes of distances to DNA are concerned, the second of the two sequence motifs strictly conserved in Dsup and Dsup-like (262–275) is closer to DNA than the first motif (90–100) and no special features are noticed in the sequence of the nucleosome-binding domain of HMGN proteins.

### The final geometries of Dsup-DNA complexes after 100 ns MD simulations reveal that both molecules are tightly aggregated

**Figure 8:**
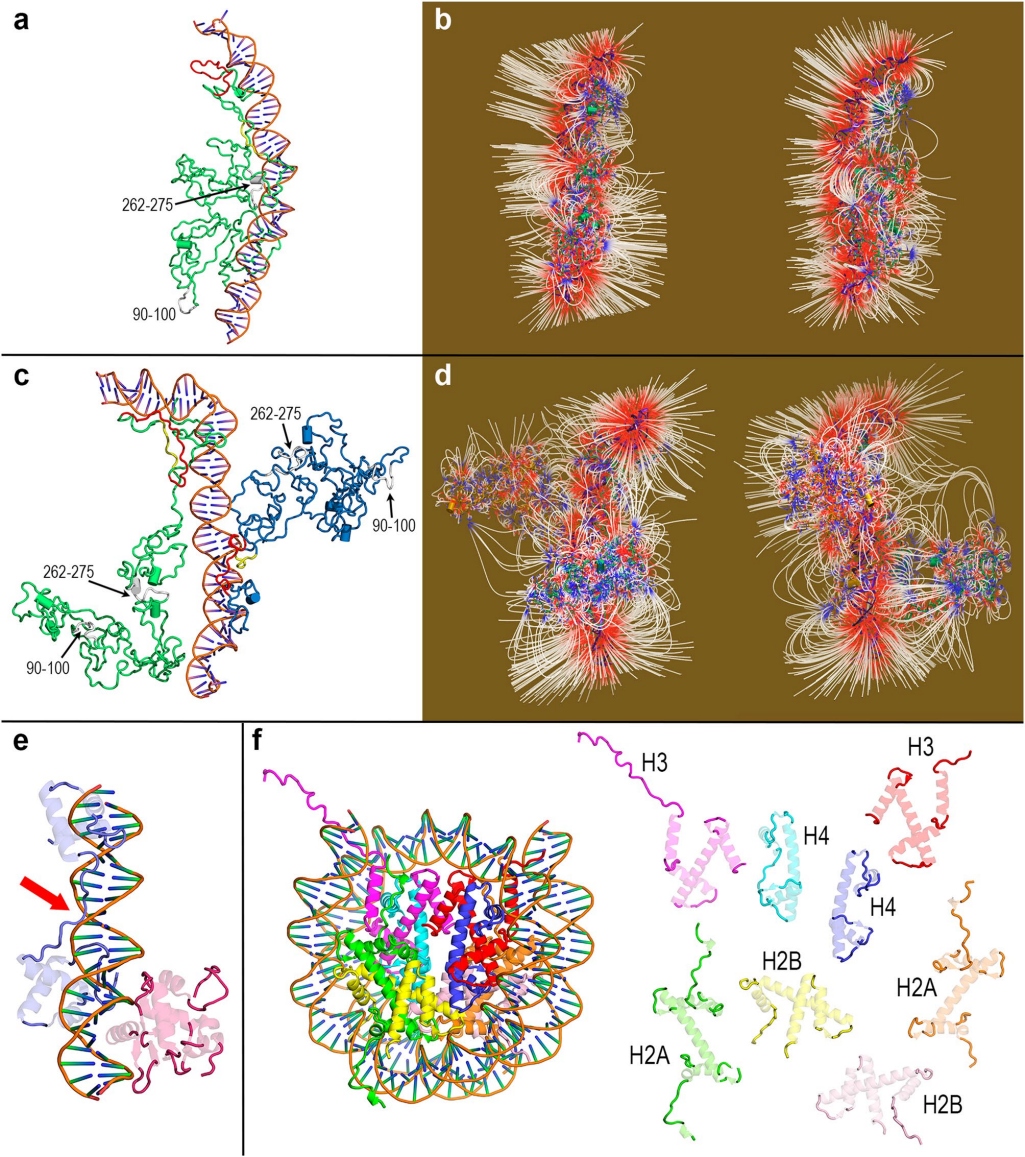
Final geometries of Dsup-DNA complexes after all-atom MD 100 ns simulations and structural disorder in DNA-binding proteins. (**a**) Dsup-DNA complex. The conserved sequence regions are marked in the protein ribbon with the same colours used in Fig. 1a. (**b**) Two views of the electric field ***E*** = 7 *kT*-equivalent units in this final geometry. (**c**) (Dsup)_2_-DNA complex. The conserved sequence regions are marked in both protein ribbons (green: DsupA, blue: DsupB) with the same colours used in Fig. 1a. (**d**) Two views of the electric field ***E*** = 7 *kT*-equivalent units in this final geometry. (**e**) Disordered regions (opaque loop segments) of the Ets-1 transcription factor (red ribbon) and its paired domain of PAX5 box protein (blue ribbon) increase DNA binding affinity by up to 1,000-fold because of their flexibility (PDB id: 1MDM). The red arrow points to the particularly long disordered segment which adapts to the DNA structure (**f**) Left: crystal structure of the nucleosome core particle of *Xenopus laevis* (PDB id: 1AOI). Wrapping DNA encloses two copies of core histones H2A, H2B, H3 and H4. Right: separate views of the eight histones at the geometry inside the nucleosome particle with same colours and disordered segments shown as opaque ribbons.

The lack of rigid structure and the high degree of dynamics endow IDPs with a flexibility to adjust their structure to binding partners, in this case DNA. In addition, the existence of long-range attraction between an IDP and its partner facilitates the formation of the so-called disordered complexes. In these complexes, it is often found that the regions flanking the interaction interface but not the interface itself remain disordered^34^. The final geometries of the two Dsup-DNA aggregates studied in this work (Fig. 8 and Supplementary Fig. 2) show features reminiscent of such disordered complexes. Although the intrinsic disorder of Dsup precludes obtaining a sharp, unique picture of its interaction with DNA, these final geometries provide some structural details of interest.

In the 1:1 complex, Dsup is tightly bound to DNA through different structural regions (Fig. 8a). The density of field lines where Dsup locates around DNA suggests an association with very strong electric fields (Fig. 8b). In the 2:1 complex, the presence of a second Dsup molecule introduces a spatial restraint in the dynamic evolution of both proteins that is noticed in the overall smaller interaction interface with respect to that found in the 1:1 complex (compare Fig. 8a, c). This notwithstanding, extended structural regions of both DsupA and DsupB show close proximity to DNA. The electric field around DNA in this 2:1 complex (Fig. 8d) shows features similar to those found in the 1:1 complex even though one might expect that the presence of a second Dsup molecule would have an effect on the electric field. In both complexes, the second of the strictly conserved motifs in Dsup (segment 262–275) and Dsup-like is much closer to DNA than the first one (segment 90–100), especially in the 1:1 complex (Fig. 8a) and in DsupB in the 2:1 complex (Fig. 8c), as already pointed out in earlier Results sections. The large mobility of a disordered structure could in principle permit electrostatic attractions between several segments of both Dsup molecules that would then be noticed in the occurrence of many electric field lines between them. However, the final geometry in Fig. 8d shows a very low number of electric field lines between the two Dsup molecules. It seems apparent that the uniformly negative electric character of DNA drives the whole interaction towards an electrostatic dominance of DNA–protein effects over possible protein–protein effects.

The abovementioned ability of IDPs to adjust their structure to that of their partners is evident in the local geometries adopted by Dsup segments closer to DNA in several interactions sites (Supplementary Fig. 2). The disordered protein backbone adjusts its local structure to fit the DNA which in turn may be bent, kinked or adopt other shapes. In this regard, it must be stressed that our MD trajectories describe properly the flexibility of DNA rapidly modifying the ideal model of B-DNA used for the initial geometry. Not only at the final structures (Figs. 8a, c, and Supplementary Fig. 2), but also along the whole simulations (Supplementary Figs. 1, 3 and 4), the molecule of DNA in fact adopts bent and kinked geometries irrespective of its interaction with the protein. In turn, the extreme flexibility of disordered Dsup allows it to adjust the fluctuating conformations of DNA because their interaction is dominated by electrostatic effects rather than by specific geometries. This structural adjustment involves interaction sites that span over significantly large segments of the protein. In all these sites, the interaction is mainly driven by the positively charged side chains of lysine and arginine residues that orient towards DNA (Supplementary Fig. 2) because of the negative charges that uniformly line DNA surface. The electrostatic complementarity between the greatest part of Dsup surface and the entire DNA surface is all the more evident when both molecules approach each other (Supplementary Figs. 3 and 4). The changes occurring when the minimum distance between Dsup and DNA decreases (Fig. 6) hint at the essential role of electrostatics in bringing together large segments of the two molecules (Supplementary Figs. 3 and 4). These changes suggest that the possible protecting role of Dsup in shielding DNA from external effects should be a consequence of the electrostatic attraction that drives the merging of the surfaces of both molecules which is also reflected into the generation of intense electric fields (Figs. 3 and 8). The flexibility of Dsup permits this shielding to act independently of DNA conformation so that from a structural point of view, its putative protecting role could be equally played either on free DNA or on circular DNA in nucleosomes.

Finally, the occurrence of secondary structure over all the MD simulations is basically restricted to transient small elements (Supplementary Table S1). These elements are on the one hand, a few small (no longer than 4 residues) α- and 3_10_-helices mainly on sequence segments ~ 100–125, ~ 160–170, ~ 270–290 and ~ 400–425, and on the other hand, a few short (3 residues) β strands scattered over the complete sequence. As far as MD results are concerned, the region ~ 140–220 for which we found that local homology-based models were predicted (Fig. 1c) and that previous in silico analyses had predicted a structured alpha helical region^14, 19^, shows no trends towards a higher content of secondary structure than the remaining regions.

## Discussion

Available evidence suggests that the tardigrade-unique Dsup protein from *R. varieornatus* and Dsup-like protein from *H. exemplaris* either directly or indirectly protect DNA from radiation damage^14, 19, 20, 24, 25^. Expression of Dsup gene in human embryonic kidney cells was observed to significantly improve cell viability and to reduce DNA strand breaks after irradiation with X-rays, compared to control cells^14, 19^. This protective role is extended to the damage caused by hydrogen peroxide^25^. Both ionising radiation and hydrogen peroxide generate hydroxyl radicals as a major ROS product in cells^35, 36^. The molecular details of the fundamental processes involved in radiation damage of DNA should address primary impact of high-energy photons and secondary particles (electrons, ions and radicals) produced by the processes following ionisation^37^. These processes can result in breaking of π-stacking and H-bond interactions or in cleavage of phosphodiester bonds, which in turn lead to distinct fragmentation pathways associated to single- and double-strand breaks^38^. Since we are still far from understanding how Dsup protein shields DNA from radiation damage, computational studies on the Dsup-DNA interaction can provide clues of potential interest to further elucidate the mechanism of this protection.

While Hashimoto et al. have shown that Dsup interacts non-specifically with free DNA^14, 19^, Chavez et al. have presented evidence that Dsup and Dsup-like also protect chromatin from cleavage by hydroxyl radicals^25^. In Refs.^14, 19^, association of Dsup with DNA was demonstrated to be prerequisite for protecting DNA from X-rays and the C-terminal region of Dsup was shown to be responsible for association with DNA as well as for co-localisation with nuclear DNA in transfected cells. Subcellular localisation prediction tools used by Hashimoto et al. suggested nuclear localisation for Dsup and Dsup-like proteins and a putative nuclear localisation signal was also identified in the C-terminal domain of both proteins. Sequence-based in silico analyses predicted secondary structure only at a middle region and also revealed that Dsup is a highly basic protein, especially in its C-terminus. Those authors suggested that the electrostatic attraction between the positive charge of Dsup and the negative charge of DNA would drive the Dsup-DNA association and conjectured that the N-terminal and middle regions of Dsup could play a role to relieve the possible adverse effects induced by formation of large Dsup-DNA complexes^14, 19^.

Chavez et al. tested the binding of Dsup to reconstituted mononucleosomes and found that Dsup binds with a significantly higher affinity to nucleosomes than to free DNA and that this binding is independent of the DNA sequence^25^. These authors showed that Dsup also binds to extended nucleosome arrays similarly to separate nucleosomes and found that the addition of the major nucleosome-binding linker histone H1 does not alter the chromatin assembly demonstrating that Dsup and histone H1 can bind simultaneously to nucleosomes. The protection against damaging effects of hydroxyl radicals was tested by these authors in free DNA and in chromatin. Their results suggested that the Dsup protection was stronger with chromatin than with free DNA which led the authors to speculate that the protein could possibly bind specifically to nucleosomes creating a highly resistant structure. Chavez et al. also identified in the C-terminus of Dsup and Dsup-like a sequence motif critical for the binding of HGMN proteins to nucleosomes and demonstrated its importance for nucleosome-binding required to protect chromatin from hydroxyl radical-mediated DNA damage^25^.

Our computational study suggests that disorder is paramount in the Dsup-DNA interaction as it endows the protein with a high flexibility to adapt its structure to DNA. The weak selective pressure associated to IDPs in which amino acids in disordered segments can change without the constraint to maintain a definite structure, together with the evolutionary adaptation to different environments as *R. varieornatus* is terrestrial while *H. exemplaris* is aquatic, could be the reasons why Dsup and Dsup-like sequences have very low identity whereas they keep the essential disorder-encoding pattern. Intrinsic disorder in full proteins as well as in domains and regions is particularly frequent in DNA and RNA binding^39, 40^. Disordered N-terminal tails are known to contribute to efficient DNA scanning^41^ and disordered C-terminal tails are often present in transcription factors and in proteins associated with DNA binding activity^42^. For instance, it has been reported that the disordered regions of the Ets-1 transcription factor^43^ (Fig. 8e) increase DNA binding affinity by 100–1,000 fold because of the flexibility of the binding segment^44^. Disorder is also very common in the histone family. The C-terminal domain of the major nucleosome-binding linker histone H1 has disordered segments that are involved in chromatin formation through binding linker DNA between the nucleosomes^45^. Tails of core histones H2A, H2B, H3 and H4 which associate with DNA to form the nucleosomes, are also intrinsically disordered regions^46–49^ (Fig. 8f). It has been proposed that the high degree of disorder allows core histones to function as switches regulating a variety of genetic processes^50^ and the computational analysis of more than 2,000 histones revealed that the majority of them have large disordered regions^51^.

The MD results presented here suggest that disordered Dsup and DNA approach each other due to the electrostatic attraction between the abundant positive charges of basic amino acids and the negative charges of phosphates. This attraction is reflected into the electric field between Dsup and DNA that show features of considerable strength even at large distances. The electrostatic complementarity between most of the Dsup interacting surface and the entire DNA surface becomes stronger when both molecules are closer together. Provided that the attraction between opposite charges takes place, the non-specificity of the Dsup-DNA association pointed out in the literature^14, 19, 25^ must be interpreted as occurring regardless the particular amino acid and nucleotides sequences. Spatial proximity to DNA modulates the disordered motion of large regions of Dsup that adjust their local structures to fit the DNA shape (see the animation of MD trajectories for Dsup-DNA 1:1 and 2:1 complexes in Supporting videos 1 and 2, respectively). This is particularly evident in the C-terminal domain that presents significantly large segments in close contact with DNA. This C-terminal region has been previously shown to be required and sufficient for DNA binding^14, 19^, which agrees with our findings that demonstrate that it invariably is the region of Dsup closest to DNA in the systems studied. Also, the dynamics of this C-terminal domain alone gives a picture of the balance between motion and electrostatic effects at play in the Dsup-DNA interaction. As mentioned, it is worth emphasising that putative sequence motifs of both nuclear localisation signal^14, 19^ and nucleosome-binding domain of HMGN proteins^25^ have been identified in the C-terminal segment. It seems thus evident that nuclear localisation and nucleosome binding critically depend on the location of sequence recognition motifs in this C-terminal domain. As for the two sequence motifs strictly conserved between Dsup and Dsup-like, our results reveal that they flank a middle sequence segment for which homology-based structural α-helical models were predicted. The MD calculations on the Dsup-DNA complexes indicate that the second motif, which is associated to a cluster of positively charged residues in the structural models of both proteins, is much closer to DNA than the first motif in Dsup. Even though our MD results reveal no particularly relevant features for these motifs, one might speculate that they could play a kind of “switching” role affecting a possible structural change from disorder to order in the middle segment mentioned. Given that the second motif is a cluster of positive charges, that switching could be activated upon union to DNA.

The lack of a rigid architecture and the high flexibility enable Dsup to adjust its structure to DNA in a shielding-like association regardless of conformational changes in some DNA regions. This detail could be relevant to explain the observed protecting role of Dsup to both free DNA and nucleosomes. Our results suggest that the high flexibility would permit Dsup to fit the bent structure of the discoidal DNA that wraps around core histones in nucleosomes (Fig. 8f). Our MD data also reveal the merging of large surface segments of Dsup and DNA when they are brought close together as a consequence of their mutual electrostatic attraction, a detail that could also be worth to consider in the quantitative elucidation of the protection mechanism.

To summarise, the computational study presented in this work lends support to previous suggestions that Dsup associates with DNA in a non-specific manner, physically shielding DNA from direct radiation and ROS damage, and also leaves room for addressing Dsup-nucleosome interactions. The picture that emerges from our results is that the intrinsic disorder of Dsup and the strong electrostatic attractions between the protein and DNA drive the formation of flexible aggregates with Dsup and DNA closely associated. One is tempted to speculate that Dsup should protect DNA from radiation and ROS damage by providing not only structural support but also electrical shielding. The inherently high flexibility of this shield would allow the complex to absorb the impact of energy and particles that should produce DNA damage by increasing the motion of large segments of Dsup.Even though intrinsic disorder precludes obtaining a unique picture of the interaction mechanism of Dsup, because an IDP exists as an ensemble of conformations that varies with time, the dynamic evolution of its structure along its attraction to DNA provides information of potential interest. While the precise mechanism of DNA protection by the tardigrade-unique Dsup protein remains to be elucidated, we believe that our computational study could represent a piece of information in this endeavour.

## Methods

### Structures

Model structures of Dsup and Dsup-like were predicted with I-TASSER^52^ from their sequences identified in the genomes of tardigrades *R. varieornatus* and *H. exemplaris*, respectively. The best I-TASSER models having C-scores = − 1.02 and − 2.65 were selected for Dsup and Dsup-like, respectively. This is a confidence score for estimating the quality of predicted models typically in the range of [− 5, 2] with higher values meaning a model with higher confidence. Structure prediction was also tried with the SwissModel^53^ server in order to search for possible sequence segments susceptible of homology-modelling. The initial model structure of DNA in the most common B conformation was prepared with the 3D-DART web^54^ from the randomly generated 57-nucleotide sequence: TTCTTGGCAACGTAACGTCAAATCCATTCGGAAACGGAACCTTAGCACCGGTAACCA.

No initial 5´and final 3´end nucleotides were assumed as we intended to simulate a continuous DNA fragment. The initial structures of the Dsup-DNA and (Dsup)_2_-DNA complexes were then modelled computationally with Chimera 1.13^55^ according to the requirements explained in the second subsection of Results. Structural disorder was predicted from the Dsup sequence with the following methods: IUPred2A^56^, PONDR-VLXT^57^, DisEMBL^58^ and DISOPRED3^59^. Analyses of structures, rendering of molecular graphics and calculation of geometrical properties were achieved with Chimera 1.13^55^, PyMOL 2.3.2 (The PyMOL Molecular Graphics System, version 2.0; Schrödinger, LLC: New York, NY, USA, 2017) and VMD 1.9.3^60^.

### Poisson–Boltzmann (PB) electrostatic potentials (EPs) and electric field

Input PQR files needed to obtain PB-EPs were firstly prepared in automated mode with the PDB2PQR server^61^. These files were then modified with in-house scripts to correct local inaccuracies in automated assignment of charges to nucleotide atoms in the modelled DNA and to test that total charges + 23 in Dsup and − 57 in DNA were obtained upon adding all the total atomic charges. With these PQR files, PB-EPs were computed solving the nonlinear PB equation with the APBS 1.5 program^62^ implemented as plug-in in PyMOL 2.3.2. Sequential focusing multigrid calculations in 3D grids with step size between 0.67 and 1.0 Å at 0.150 M NaCl concentration and temperature 298 K were performed at initial and final geometries as well as at the frames in the MD trajectories of Dsup-DNA and (Dsup)_2_-DNA complexes analysed in “Results”. With those step sizes, the space grids with the numerical output of the PB-EPs *V*(**r**) ranged from 161 × 161 × 289 = 7,491,169 points to 449 × 225 × 289 = 29,196,225 points depending on the dimensions of the system at the different frames studied. This numerical output of *V*(**r**) was saved in OpenDX scalar format for further mapping onto molecular surfaces and calculation of 3D isopotential surfaces. These DX files were also used to compute the electric field ***E*** = − *V*(**r**) with VMD 1.9.3^60^. *V*(**r**) is given in unis of *kT*/*e*, *k* being Boltzmann’s constant, *T* absolute temperature 298 K, and *e* unit electron charge.

### All-atom molecular dynamics (MD) simulations

All-atom MD simulations were run over simulation times of 100 ns with the CHARMM 3.6 force field^63^ and the c36m parameter set that has improved accuracy for treating intrinsically disordered proteins^64^. MD calculations were performed with the high performance computing Power-MPI version of NAMD 1.12^65^ in the Magerit3 supercomputer of Universidad Politécnica de Madrid. All isolated protein and protein-DNA systems were initially prepared using the PDB Reader service of the CHARMM-GUI server^66^. Periodic solvation boxes were constructed with 15 Å spacing and water molecules according to the TIP3P model^67^. Sodium and chloride ions were added to counter total charges of the protein and protein-DNA systems while setting 0.150 M salt concentration. Particle-mesh Ewald summation method^68^ was used for long-range electrostatics and a 10 Å cutoff was set for short-range non-bonded interactions. Initial geometries in all systems were minimised at 5,000 conjugate-gradient steps after which water was equilibrated at 298 K and 1 atm for 100 ps at 2 fs time steps. Production runs were then performed for 100 ns at 2 fs time steps (50 million steps per calculation) in the NPT ensemble at P = 1 atm and T = 298 K. Langevin dynamics for T control and Nosé-Hoover Langevin piston method for P control were used. NAMD output was stored every 20,000 steps thus rendering trajectories composed of 2,500 frames which were processed and analysed with VMD 1.9.3^60^ and Carma 1.7^69^.

## Supplementary information

Supplementary Information.

Supplementary Video 1.

Supplementary Video 2.
