# Supplementary material for: A computational structural study on the DNA-protecting role of the tardigrade-unique Dsup protein
Source: Sci Rep. 2020 Aug 7;10:13424. doi: 10.1038/s41598-020-70431-1 (PMC7414916; doi:10.1038/s41598-020-70431-1)
Supplement: Supplementary file 1 — Supplementary Information. [file 41598_2020_70431_MOESM1_ESM.docx]

**A computational structural study on the DNA-protecting role of the tardigrade-unique Dsup protein**

Marina Mínguez-Toral, Bruno Cuevas-Zuviría, María Garrido-Arandia &

Luis F. Pacios^*^

**Supplementary Information**

Page

Supplementary Figure 1 S2

Supplementary Figure 2 S3

Supplementary Figure 3 S4

Supplementary Figure 4 S5

Supplementary Table S1 S6

Information on supplementary videos 1 and 2 S8

**
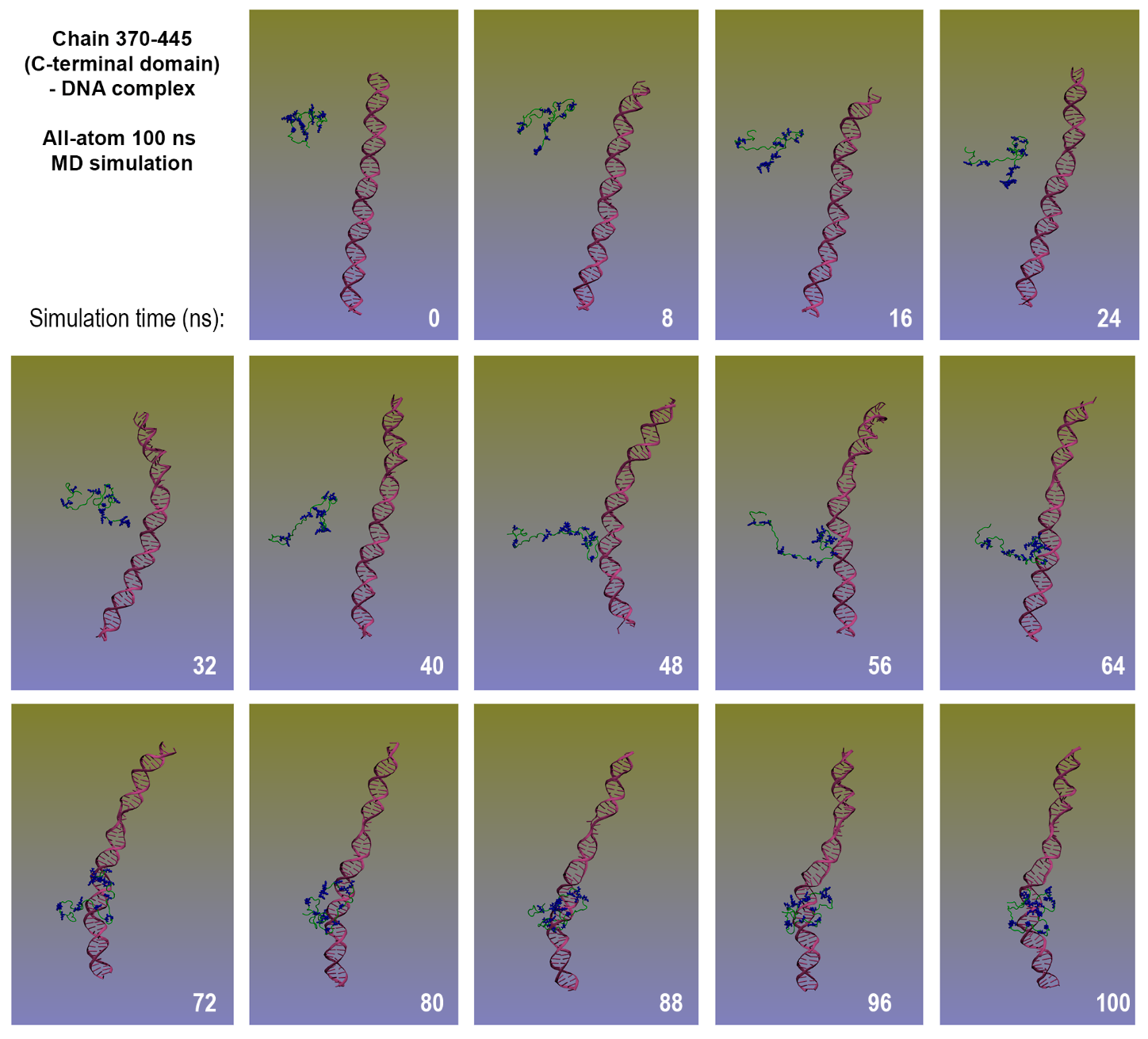
**

**Supplementary Fig. 1. Snapshots of all-atom 100 ns MD simulation of the C-terminal domain of Dsup alone in complex with DNA.** The C-terminal domain chain is depicted as a green ribbon with the positively charged amino acids shown as blue balls.

**
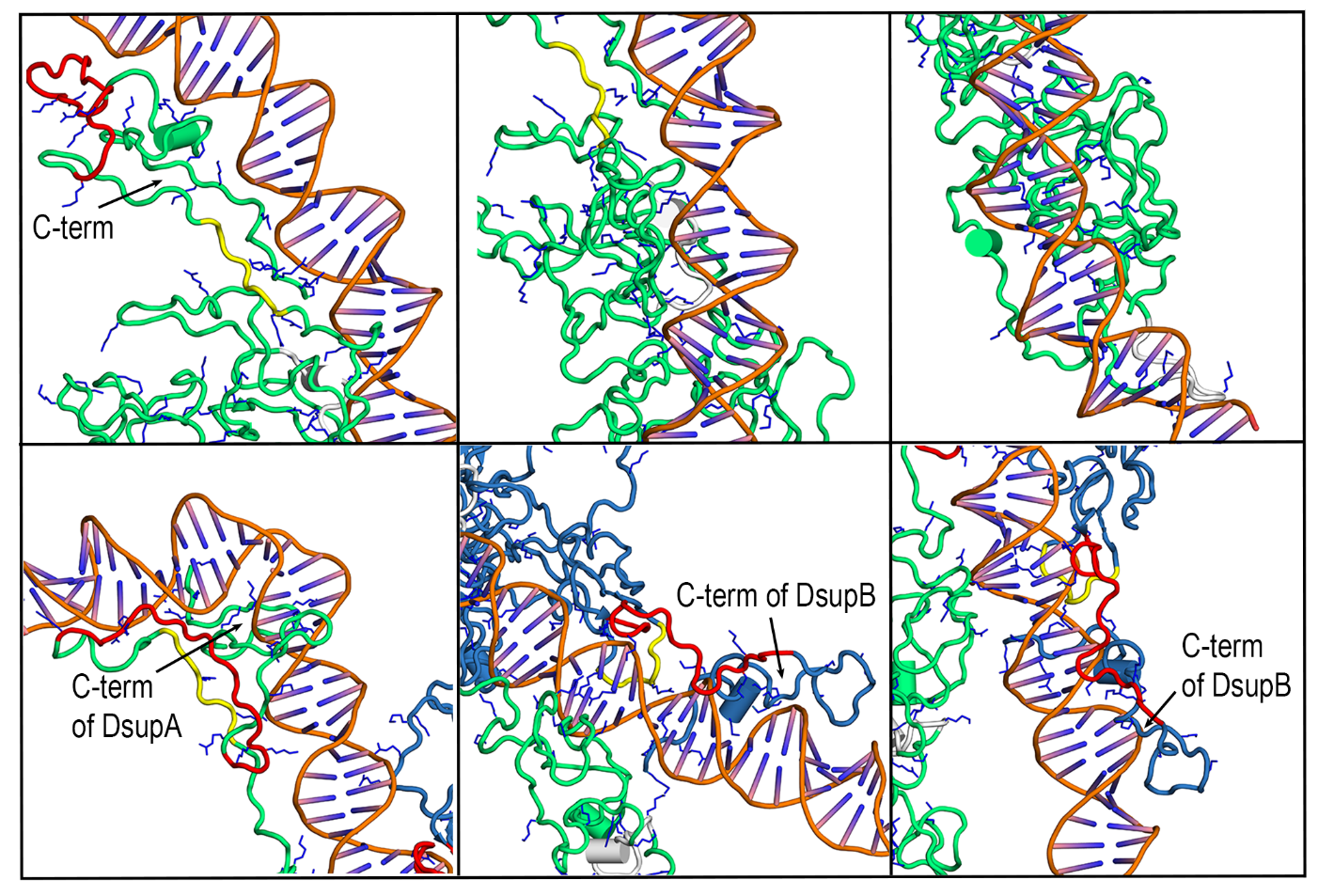
**

**Supplementary Fig. 2. Structural detail of Dsup-DNA interaction sites in the final geometries of Dsup-DNA complexes after all-atom MD 100 ns simulations.** Structural regions of the interaction between Dsup and DNA. Upper row: views of top, middle and bottom (left to right) sites of the Dsup-DNA complex at the orientation shown in Fig. 8a. Lower row: views of top, middle and bottom (left to right) sites of the (Dsup)_2_-DNA complex at the orientation shown in Fig. 8c.

**
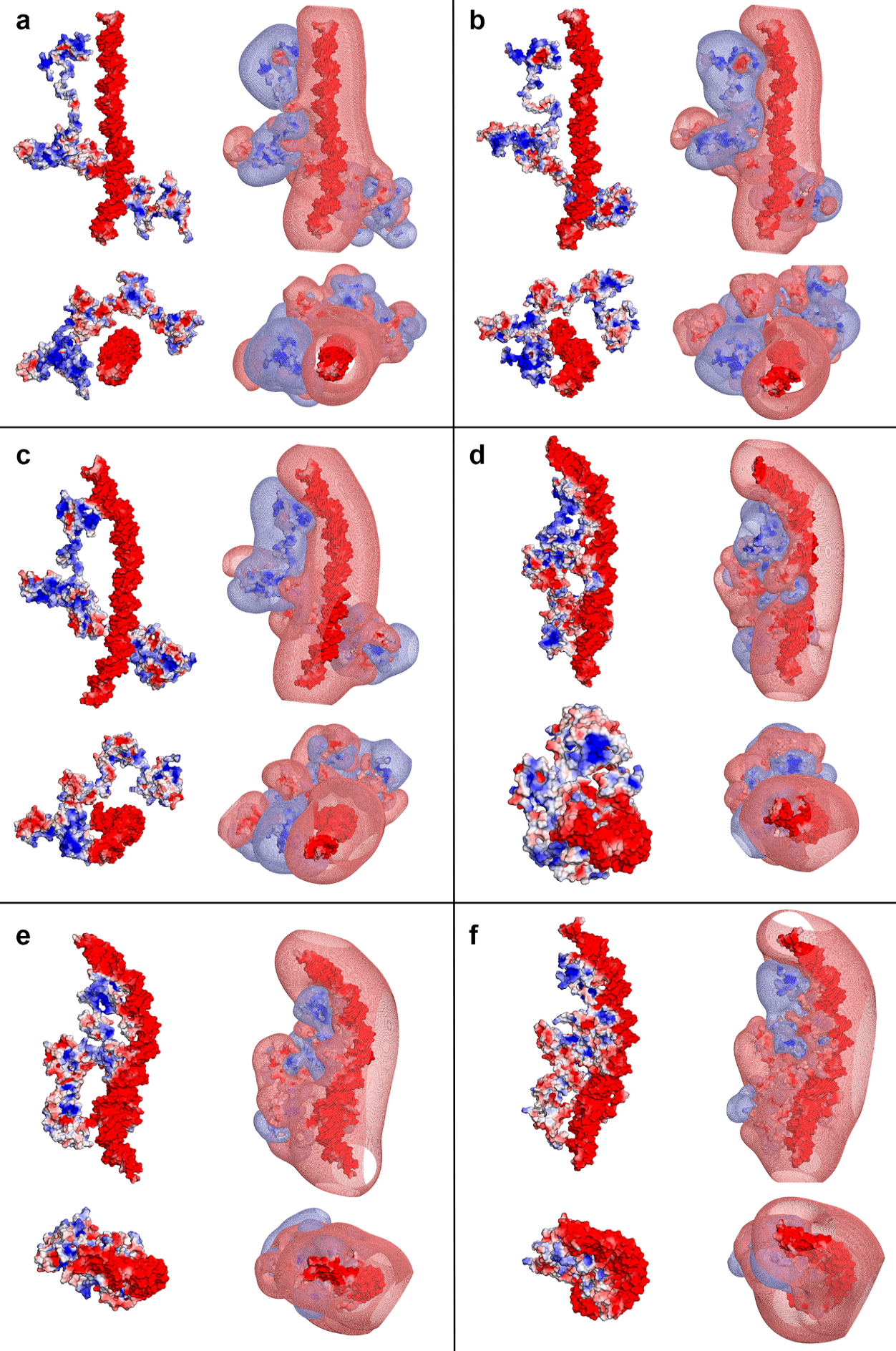
**

**Supplementary Fig. 3. Poisson-Boltzmann electrostatic potential in six snapshots of all-atom MD simulation of the Dsup-DNA complex.** The first 3 snapshots correspond to simulation times before and after the decrease of the minimum Dsup-DNA distance (*d*_min_) in Fig. 6a. The other 3 snapshots correspond to 50%, 80% and 100% total simulation time. (**a**) 4 ns, *d*_min_ = 12 Å. (**b**) 8 ns, *d*_min_ = 9.9 Å. (**c**) 10 ns, *d*_min_ = 4.2 Å. (**d**) 50 ns, *d*_min_ = 4.0 Å. (**e**) 80 ns, *d*_min_ = 3.9 Å. (**f**) 100 ns, *d*_min_ = 3.9 Å. The upper row in each panel shows side views and the lower row shows top views of the structure. The left column in each panel shows the PB-EP mapped onto the surfaces of Dsup and DNA and the right column shows the +0.1 (blue) and -0.1 (red) isosurfaces of the PB-EP in *kT*/*e* units.

**
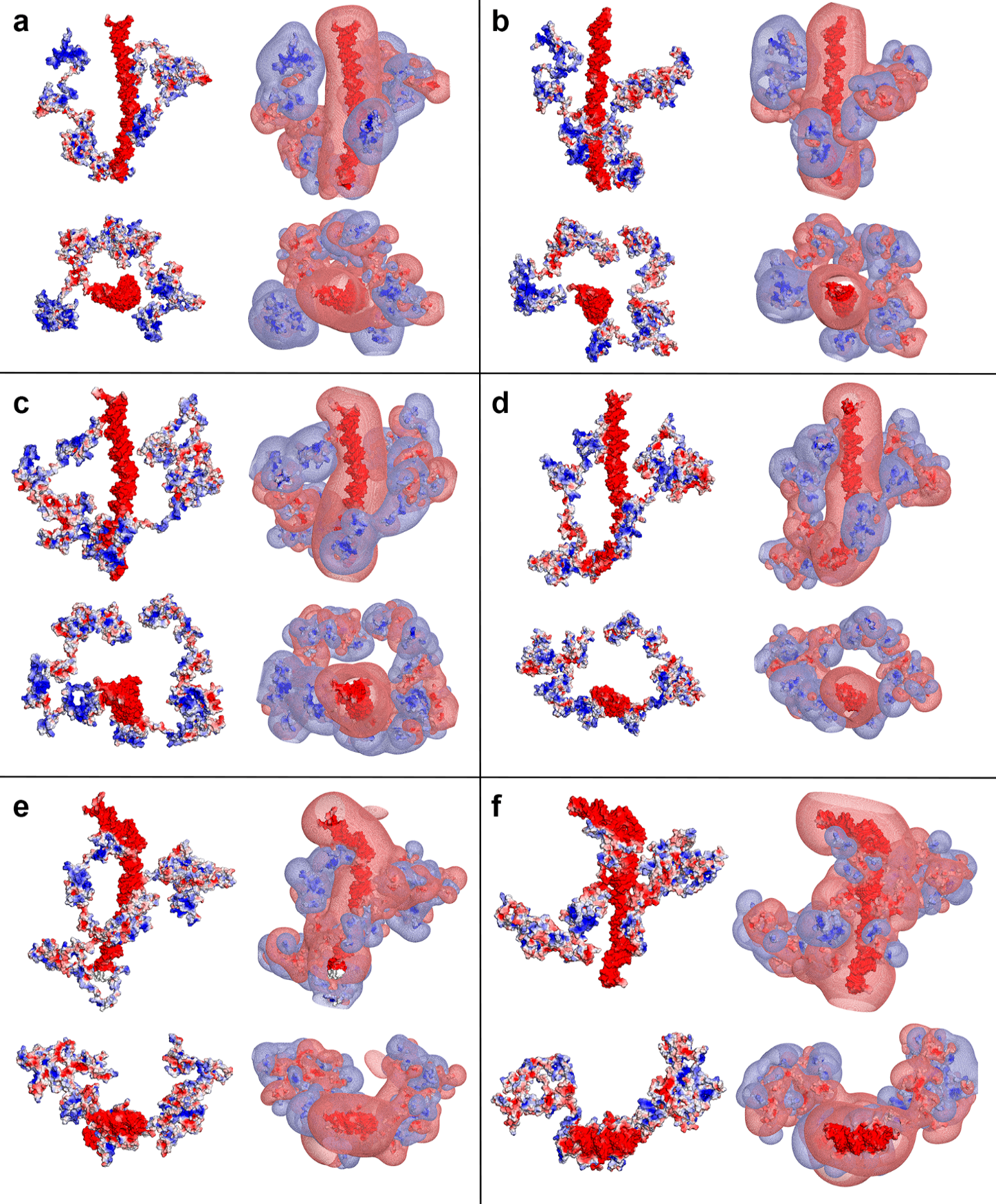
**

**Supplementary Fig. 4. Poisson-Boltzmann electrostatic potential in six snapshots of all-atom MD 100 ns simulation of the (Dsup)_2_-DNA complex.** The first 3 snapshots correspond to simulation times before and after the decrease of minimum DsupA-DNA and DsupB-DNA distances (*d*_min,A_ and *d*_min,B_, respectively) in Fig. 6a. The other 3 snapshots correspond to 50%, 80% and 100% total simulation time. (**a**) 32 ns, *d*_min,A_ = 17 Å, *d*_min,B_ = 13 Å. (**b**) 34 ns, *d*_min,A_ = 19 Å, *d*_min,B_ = 4.5 Å. (**c**) 38 ns, *d*_min,A_ = 5.0 Å, *d*_min,B_ = 4.4 Å. (**d**) 50 ns, *d*_min,A_ = 4.1 Å, *d*_min,B_ = 4.2 Å. (**e**) 80 ns, *d*_min,A_ = 4.1 Å, *d*_min,B_ = 4.1 Å. (**f**) 100 ns, *d*_min,A_ = 3.9 Å, *d*_min,B_ = 3.9 Å. In all cases, DsupA and DsupB molecules are shown at the left and at the right of DNA, respectively. The upper row in each panel shows side views and the lower row shows top views of the structure. The left column in each panel shows the PB-EP mapped onto the surfaces of both Dsup proteins and DNA and the right column shows the +0.1 (blue) and -0.1 (red) isosurfaces of the PB-EP in *kT*/*e* units.

**Table S1. Elements of secondary structure identified with DSSP in selected frames of the all-atom MD 100 ns trajectories computed in this work.** For isolated Dsup trajectories, only initial, middle and final frames are given. For Dsup in complex with DNA, the frames listed correspond to those displayed in Supplementary Figures 3 and 4 for 1:1 and 2:1 complexes, respectively .

Frame Element Sequence segments

Isolated Dsup in trajectory 1

t = 0 ns 3_10_ helix: 75-77 120-122 224-226 312-314

α helix: 264-267

t = 50 ns 3_10_ helix: 375-377

α helix: 26-31 40-44 165-168

β-strand: 118-119 142-143

t = 100 ns 3_10_ helix: 270-272 331-333

α helix: 122-125 165-169 187-192

β-strand: 327-328 335-336 388-389 419-420

Isolated Dsup in trajectory 2

t = 0 ns 3_10_ helix: 75-77 121-123 167-169 264-266

α helix: 335-338

t = 50 ns 3_10_ helix: 105-107 270-272

α helix: 289-292

β-strand: 63-64 68-69 280-281 285-286 403-406 410-413

t = 100 ns α helix: 166-169 289-293

β-strand: 62-64 68-70 214-215 240-241 401-402 414-415

Dsup in the 1:1 complex

t = 4 ns 3_10_ helix: 174-176

α helix: 43-46 103-107 120-123 166-169 285-288 296-299

t = 8 ns α helix: 103-107 120-123 166-175 296-299 411-414

t = 10 ns 3_10_ helix: 166-168 187-189

α helix: 103-107 120-123 296-299 414-417

t = 50 ns 3_10_ helix: 166-168 187-189

α helix: 103-107 120-123 296-299 411-414

t = 80 ns 3_10_ helix: 120-123 427-429

α helix: 166-169 270-273 411-414

t = 100 ns α helix: 166-169 270-273 411-414

β-strand: 285-286 290-291

**Table S1 continued.**

Frame Element Sequence segments

DsupA in the 2:1 complex

t = 32 ns 3_10_ helix: 311-314

α helix: 10-13 120-123 166-169 296-299 414-417

β-strand: 231-233 246-249

t = 34 ns 3_10_ helix: 39-41

α helix: 120-123 166-169 296-299

t = 38 ns 3_10_ helix: 10-12

α helix: 120-123 166-169 296-299 414-417

β-strand: 230-231 245-246

t = 50 ns 3_10_ helix: 138-140 296-298

α helix: 10-14 166-169

β-strand: 2-3 78-79 393-394 423-424

t = 80 ns 3_10_ helix: 138-140 251-253 270-273 402-404

α helix: 10-14 159-162 166-169 296-299

β-strand: 174-175 193-194 393-394 410-412 420-424

t = 100 ns α helix: 10-13 138-141 166-169 270-273 312-315

β-strand: 127-128 144-145

DsupB in the 2:1 complex

t = 32 ns α helix: 165-168 410-414

t = 34 ns 3_10_ helix: 32-34

α helix: 120-123 165-168 410-414

t = 38 ns 3_10_ helix: 422-424

α helix: 166-169 410-414

t = 50 ns 3_10_ helix: 422-424

α helix: 120-123 165-169

t = 80 ns 3_10_ helix: 433-435

α helix: 166-169 296-299

t = 100 ns 3_10_ helix: 32-34 433-435

α helix: 10-13 166-169

β-strand: 119-120 145-146 374-375 382-383


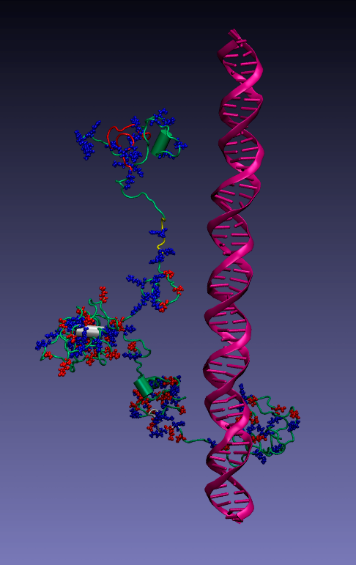


**First frame in supplementary video 1.**

This video is an animation consisting of 833 frames corresponding to one of every three frames of the 2,500-frame trajectory of the all-atom MD 100 ns simulation of the Dsup-DNA complex.

Dsup protein is shown as a green ribbon with segments that correspond to the conserved sequence motifs indicated in the text coloured as in Fig. 1a. Atoms of amino acids with positive and negative charge are depicted as VDW spheres with blue and red colours, respectively. The C-terminal region of Dsup is the upper part of the ribbon.

DNA is shown as a cartoon coloured in magenta.

Water molecules are omitted for clarity and the motion is smoothed by averaging over 20 frames in the complete 2,500-frame trajectory.

**First frame in supplementary video 2.**


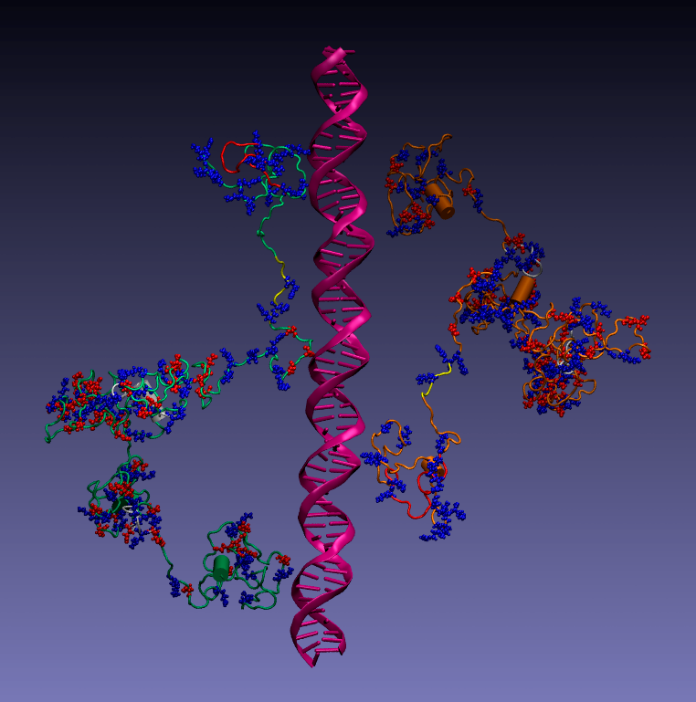
This video is an animation consisting of 833 frames corresponding to one of every three frames of the 2,500-frame trajectory of the all-atom MD 100 ns simulation of the (Dsup)_2_-DNA complex.

DsupA and DsupB molecules are shown as green and orange ribbons, respectively. In both cases, the segments that correspond to the conserved sequence motifs indicated in the text are coloured as in Fig. 1a. Atoms of amino acids with positive and negative charge are depicted as VDW spheres with blue and red colours, respectively. The C-terminal region of DsupA is the upper part of the green ribbon while the C-terminal region of DsupB is the lower part of the orange ribbon.

DNA is shown as a cartoon coloured in magenta.

Water molecules are omitted for clarity and the motion is smoothed by averaging over 20 frames in the complete 2,500-frame trajectory.
